# Supplementary figures and images for: The RgaS-RgaR two-component system promotes Clostridioides difficile sporulation through a small RNA and the Agr1 system
Source: PLoS Genet. 2023 Oct 16;19(10):e1010841. doi: 10.1371/journal.pgen.1010841 (PMC10602386; doi:10.1371/journal.pgen.1010841)

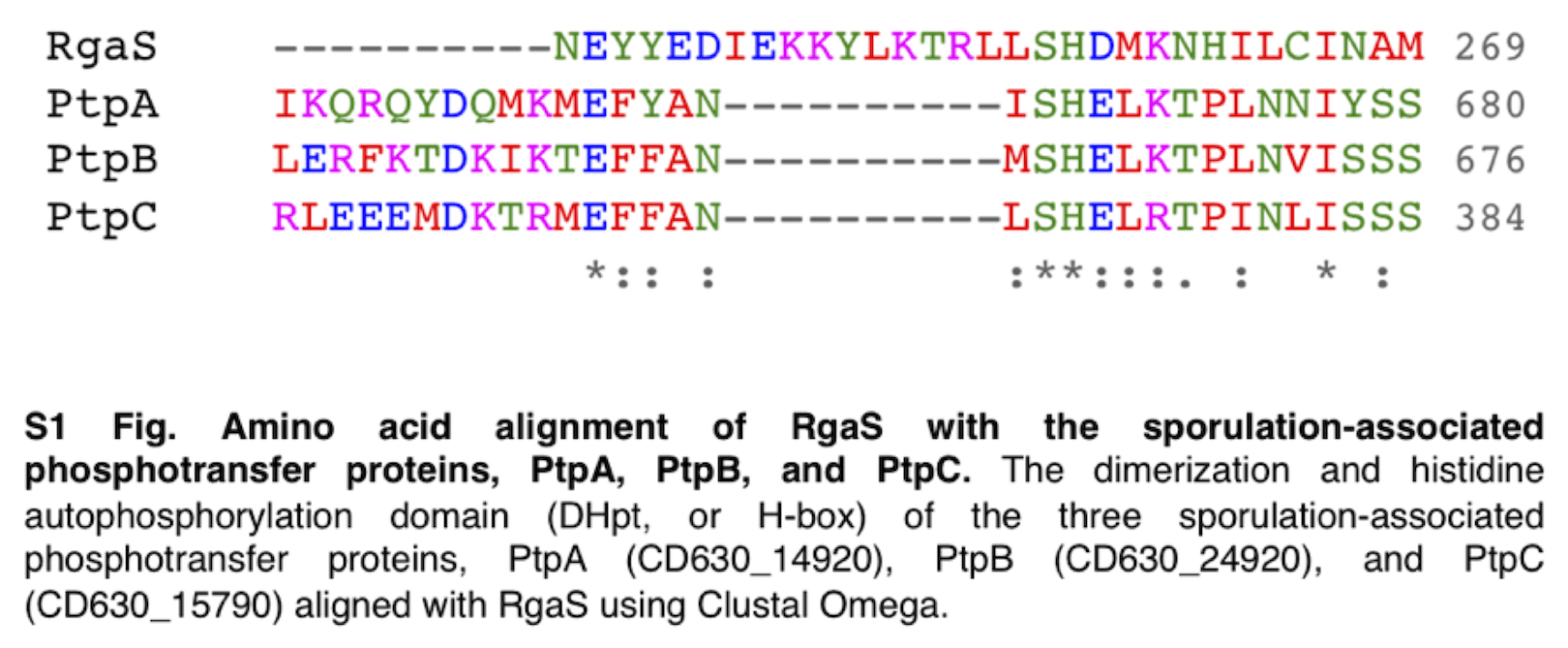

Supplement: S1 Fig — The dimerization and histidine autophosphorylation domain (DHpt, or H-box) of the three sporulation-associated phosphotransfer proteins, PtpA (CD630_14920), PtpB (CD630_24920), and PtpC (CD630_15790) aligned with RgaS using Clustal Omega. (TIFF) [file pgen.1010841.s001.tiff]

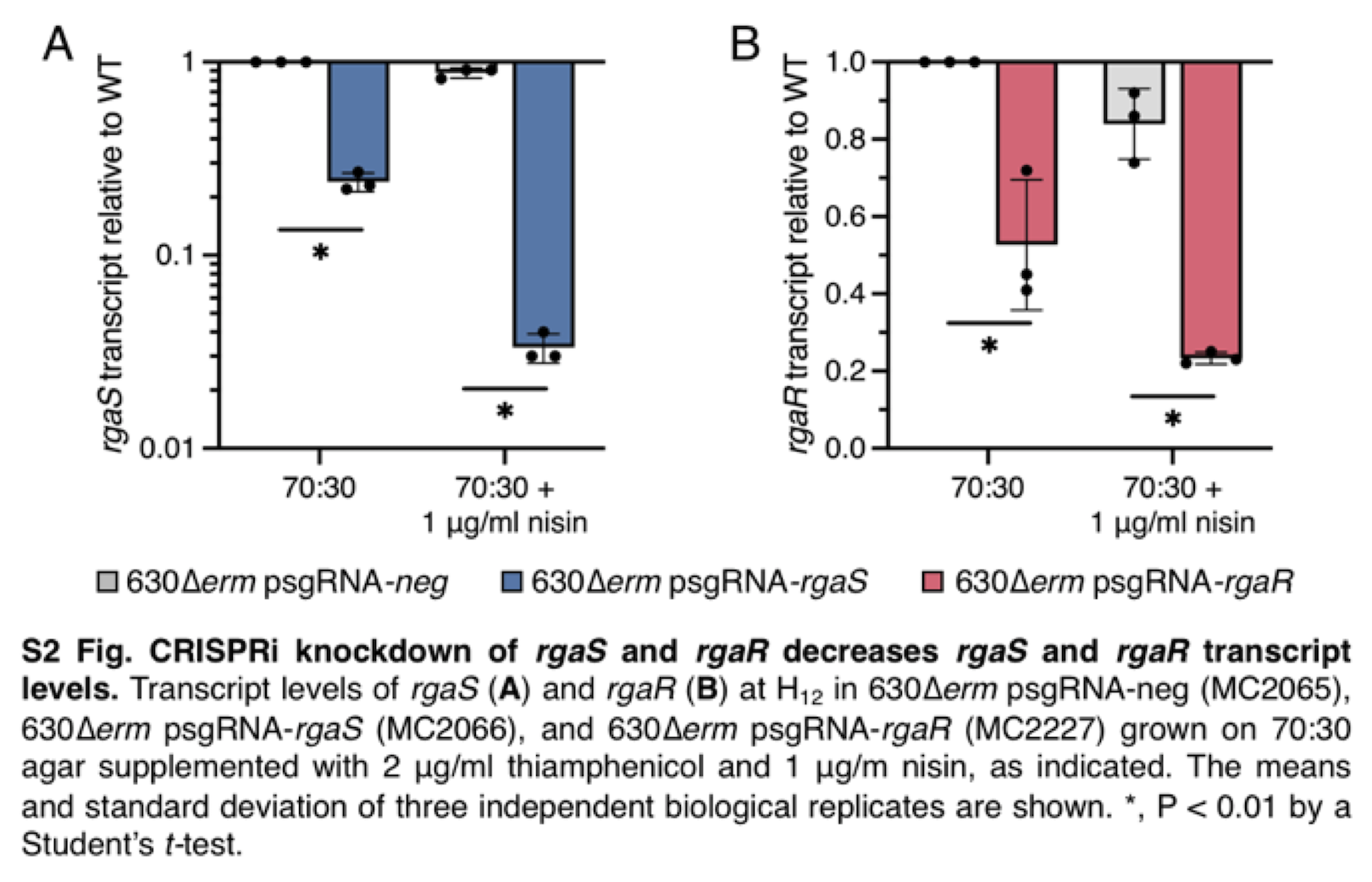

Supplement: S2 Fig — Transcript levels of rgaS (A) and rgaR (B) at H12 in 630Δerm psgRNA-neg (MC2065), 630Δerm psgRNA-rgaS (MC2066), and 630Δerm psgRNA-rgaR (MC2227) grown on 70:30 agar supplemented with 2 μg/ml thiamphenicol and 1 μg/m nisin, as indicated. The means and standard deviation of three independent biological replicates are shown. *, P < 0.01 by a Student’s t-test. (TIFF) [file pgen.1010841.s002.tiff]

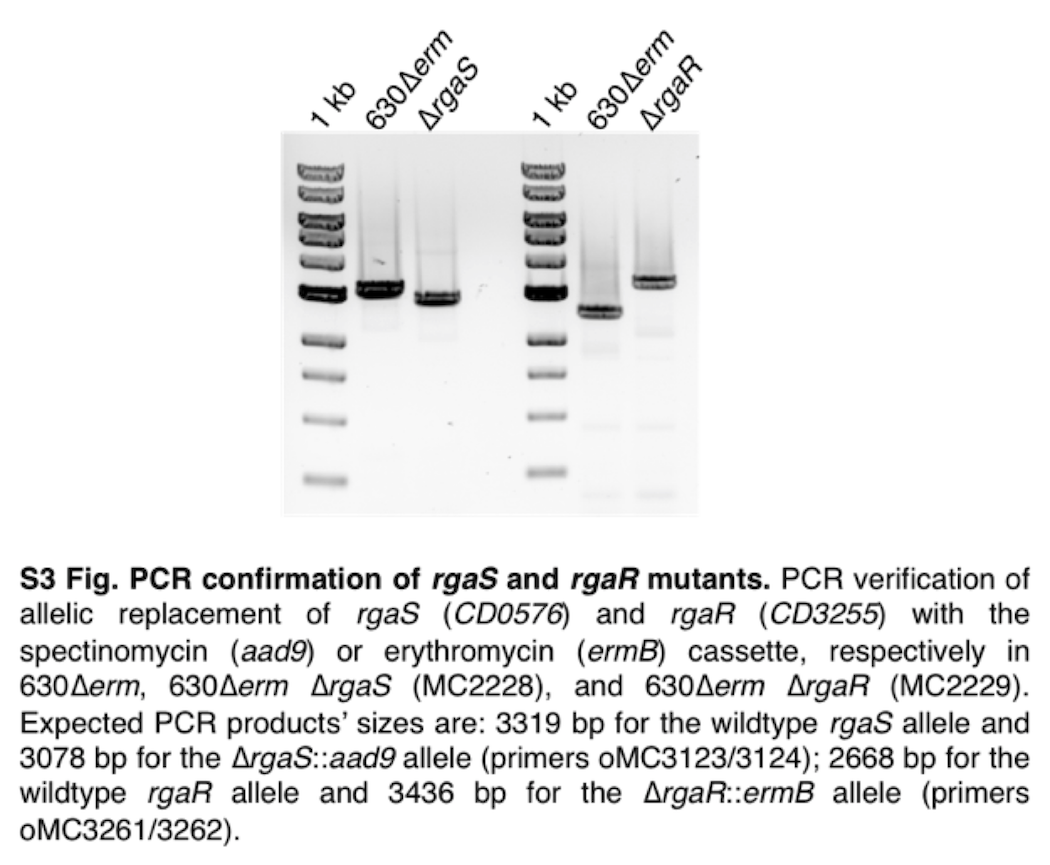

Supplement: S3 Fig — PCR verification of allelic replacement of rgaS (CD0576) and rgaR (CD3255) with the spectinomycin (aad9) or erythromycin (ermB) cassette, respectively in 630Δerm, 630Δerm ΔrgaS (MC2228), and 630Δerm ΔrgaR (MC2229). Expected PCR products’ sizes are: 3319 bp for the wildtype rgaS allele and 3078 bp for the ΔrgaS::aad9 allele (primers oMC3123/3124); 2668 bp for the wildtype rgaR allele and 3436 bp for the ΔrgaR::ermB allele (primers oMC3261/3262). (TIFF) [file pgen.1010841.s003.tiff]

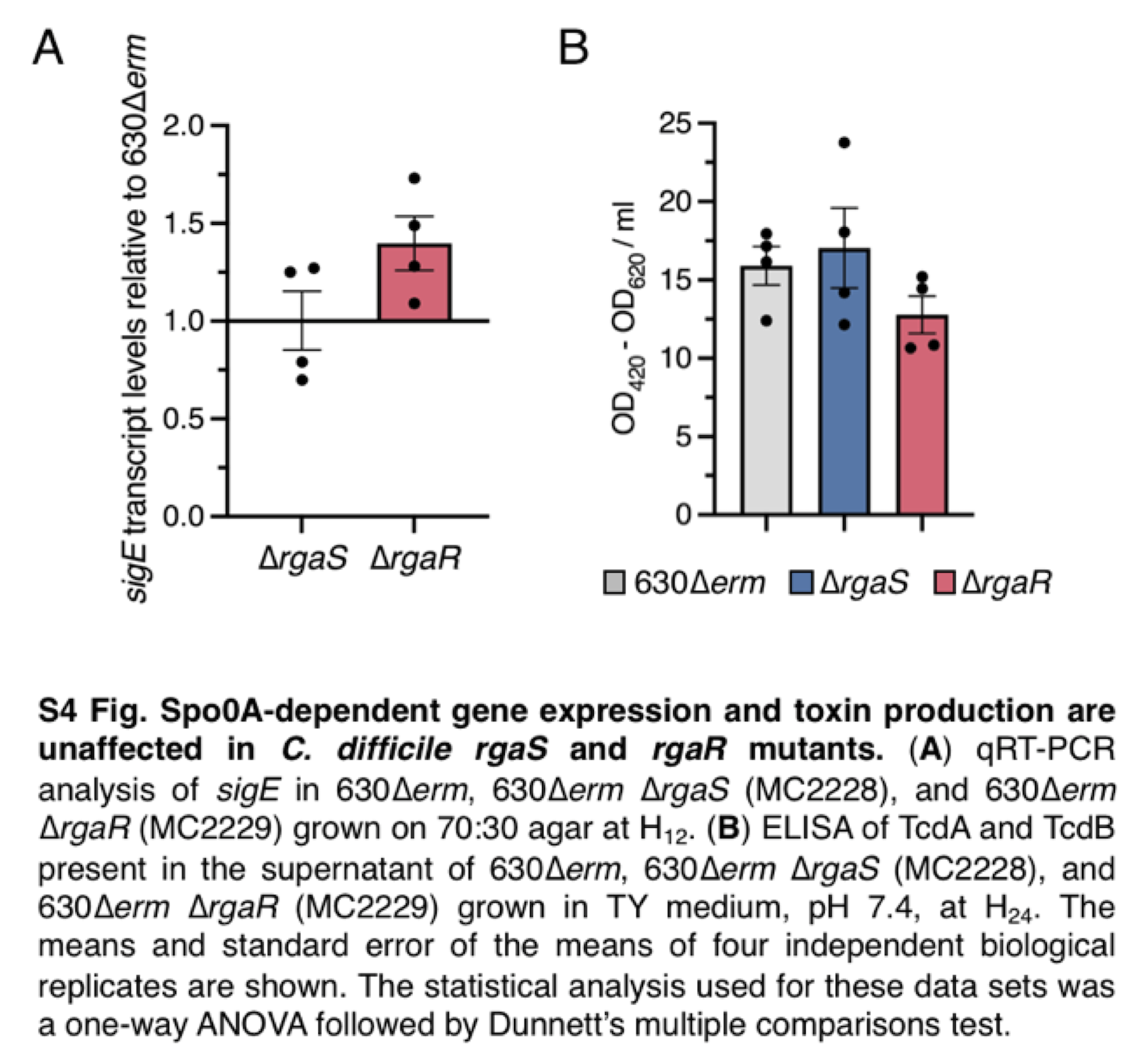

Supplement: S4 Fig — (A) qRT-PCR analysis of sigE in 630Δerm, 630Δerm ΔrgaS (MC2228), and 630Δerm ΔrgaR (MC2229) grown on 70:30 agar at H12. (B) ELISA of TcdA and TcdB present in the supernatant of 630Δerm, 630Δerm ΔrgaS (MC2228), and 630Δerm ΔrgaR (MC2229) grown in TY medium, pH 7.4, at H24. The means and standard error of the means of four independent biological replicates are shown. The statistical analysis used for these data sets was a one-way ANOVA followed by Dunnett’s multiple comparisons test. (TIFF) [file pgen.1010841.s004.tiff]

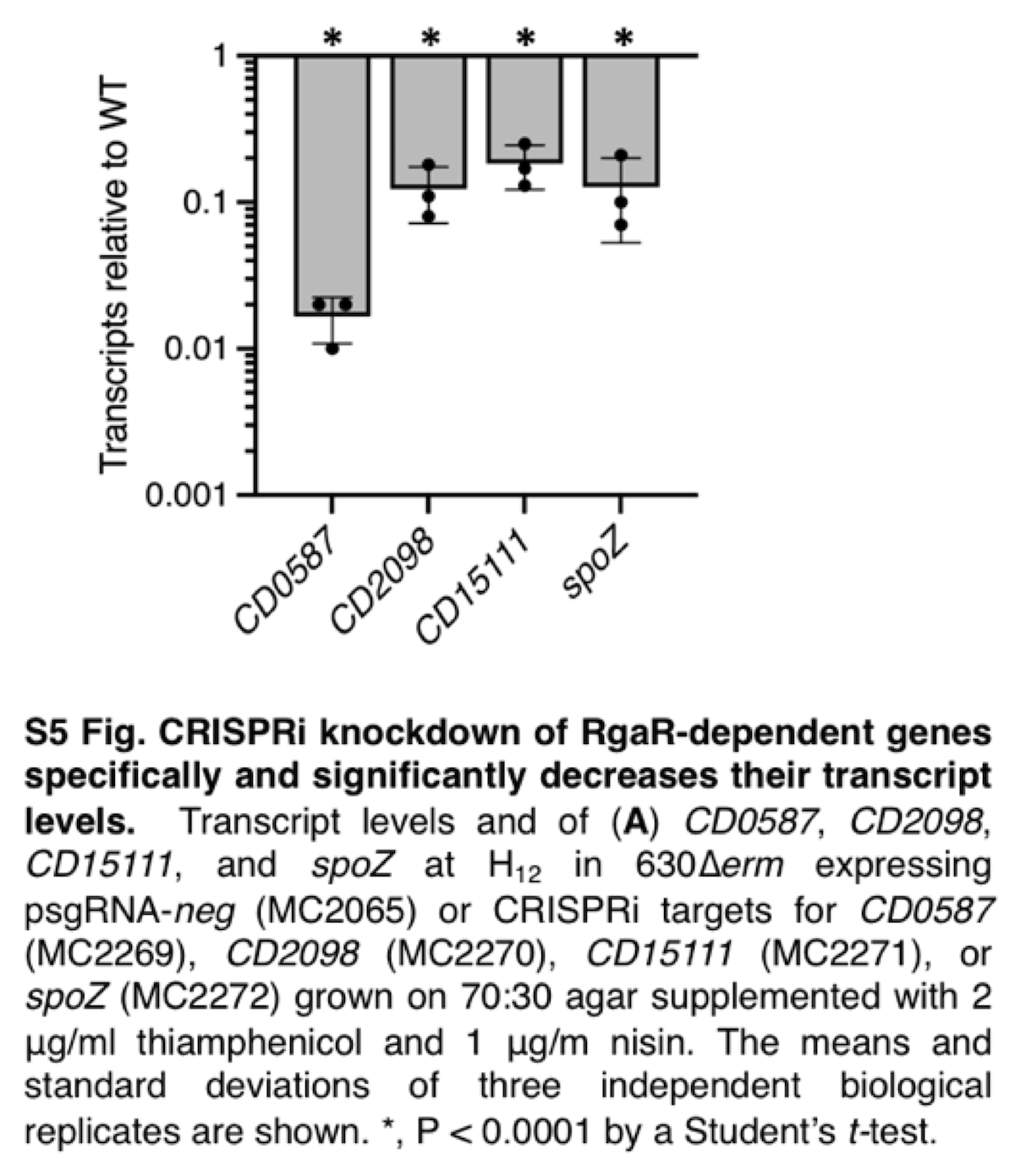

Supplement: S5 Fig — Transcript levels and of (A) CD0587, CD2098, CD15111, and spoZ at H12 in 630Δerm expressing psgRNA-neg (MC2065) or CRISPRi targets for CD0587 (MC2269), CD2098 (MC2270), CD15111 (MC2271), or spoZ (MC2272) grown on 70:30 agar supplemented with 2 μg/ml thiamphenicol and 1 μg/m nisin. The means and standard deviations of three independent biological replicates are shown. *, P < 0.0001 by a Student’s t-test. (TIFF) [file pgen.1010841.s005.tiff]

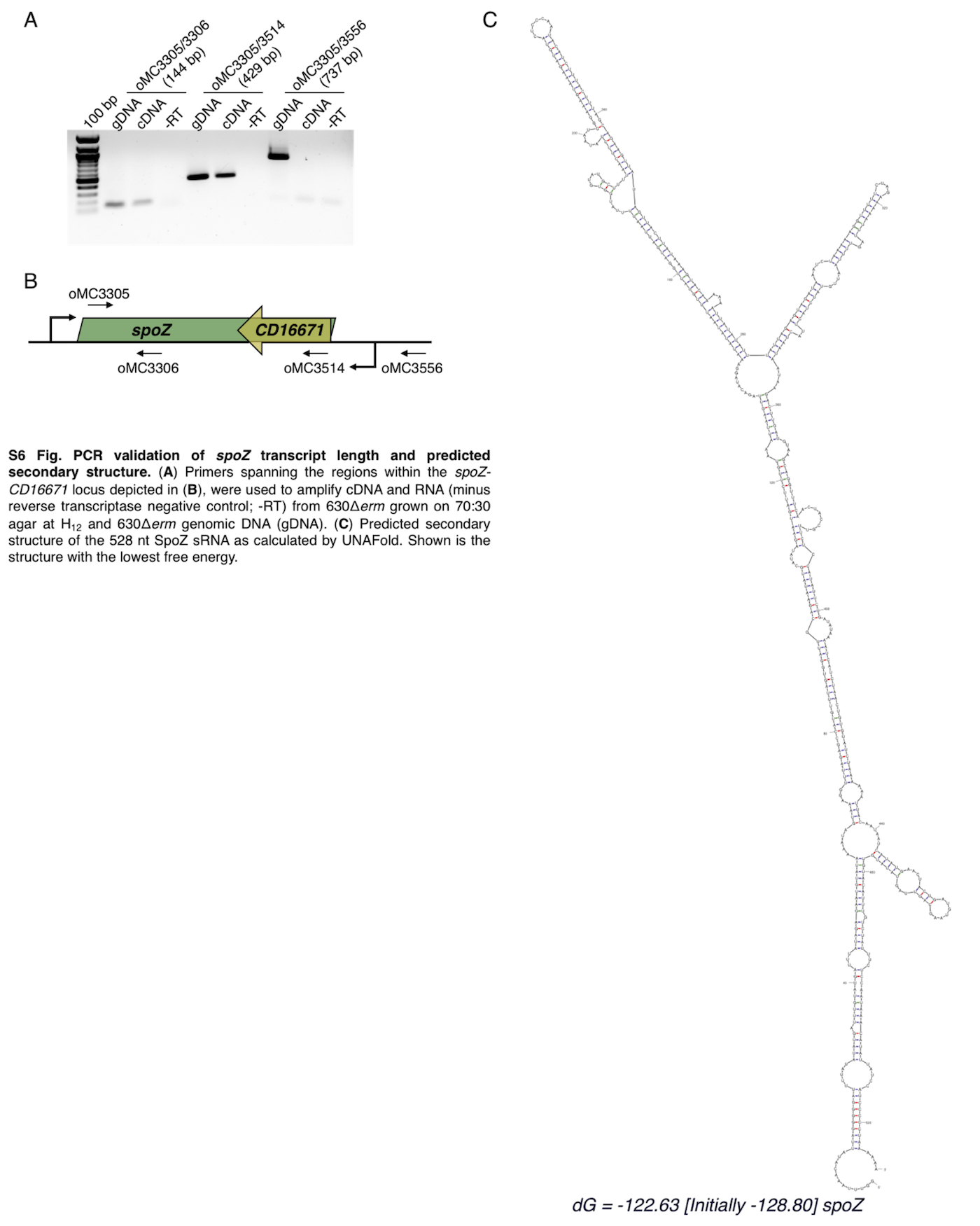

Supplement: S6 Fig — (A) Primers spanning the regions within the spoZ-CD16671 locus depicted in (B), were used to amplify cDNA and RNA (minus reverse transcriptase negative control; -RT) from 630Δerm grown on 70:30 agar at H12 and 630Δerm genomic DNA (gDNA). (C) Predicted secondary structure of the 528 nt SpoZ sRNA as calculated by UNAFold. Shown is the structure with the lowest free energy. (TIFF) [file pgen.1010841.s006.tiff]

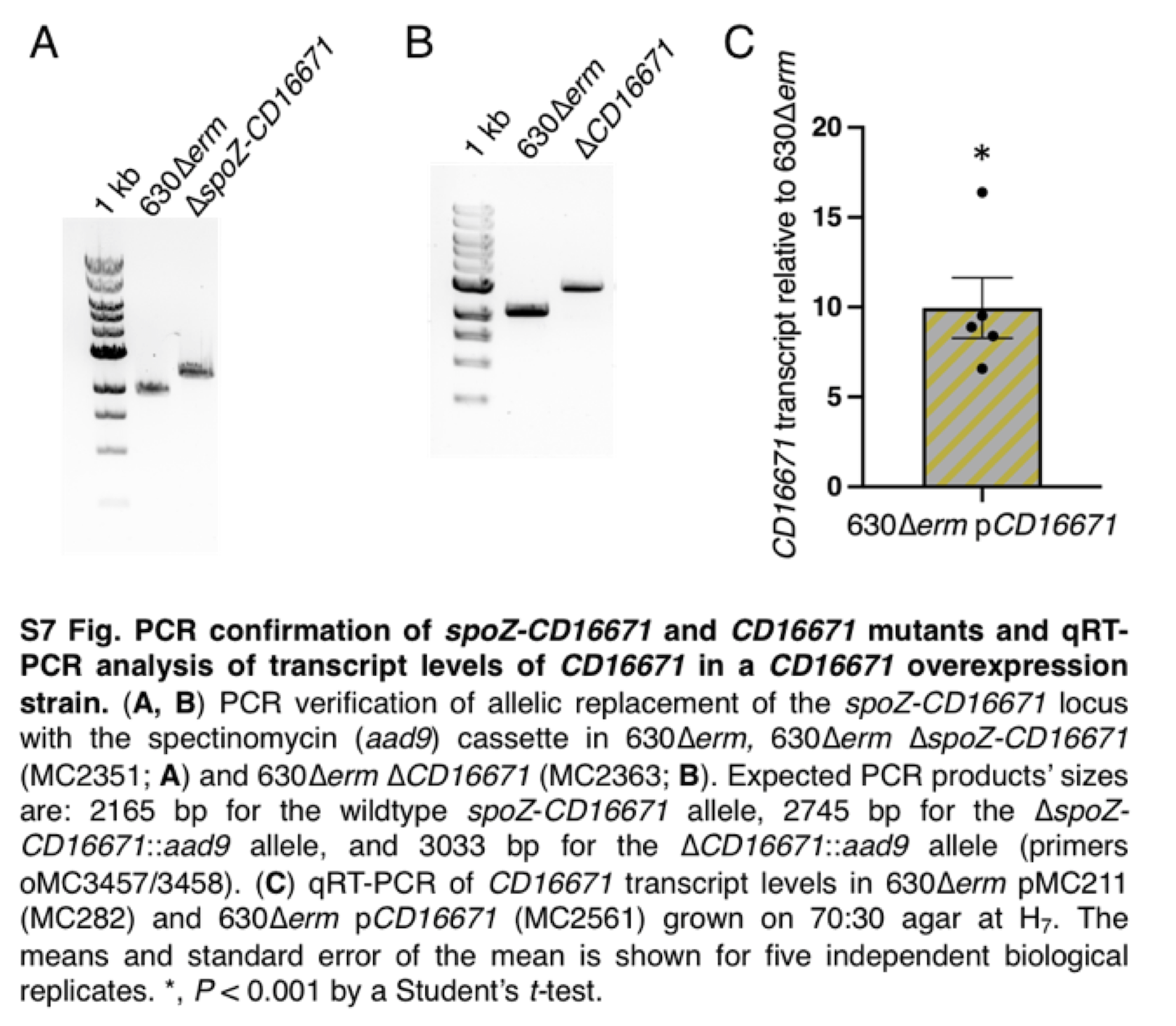

Supplement: S7 Fig — (A, B) PCR verification of allelic replacement of the spoZ-CD16671 locus with the spectinomycin (aad9) cassette in 630Δerm, 630Δerm ΔspoZ-CD16671 (MC2351; A) and 630Δerm ΔCD16671 (MC2363; B). Expected PCR products’ sizes are: 2165 bp for the wildtype spoZ-CD16671 allele, 2745 bp for the ΔspoZ-CD16671::aad9 allele, and 3033 bp for the ΔCD16671::aad9 allele (primers oMC3457/3458). (C) qRT-PCR of CD16671 transcript levels in 630Δerm pMC211 (MC282) and 630Δerm pCD16671 (MC2561) grown on 70:30 agar at H7. The means and standard error of the mean is shown for five independent biological replicates. *, P < 0.001 by a Student’s t-test. (TIFF) [file pgen.1010841.s007.tiff]

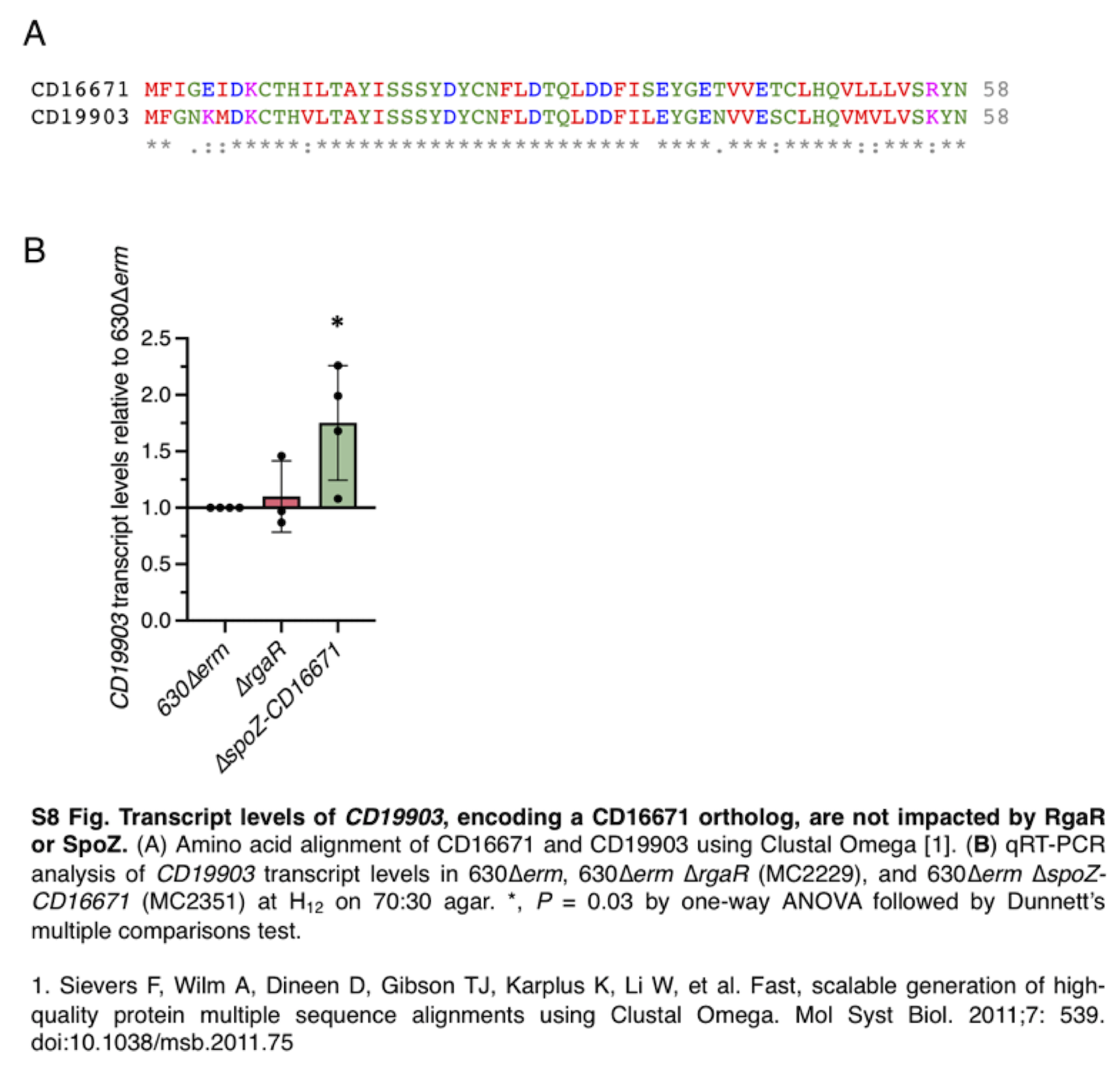

Supplement: S8 Fig — (A) Amino acid alignment of CD16671 and CD19903 using Clustal Omega (Sievers, et al. 2011 Mol Sys Biol. 7:539). (B) qRT-PCR analysis of CD19903 transcript levels in 630Δerm, 630Δerm ΔrgaR (MC2229), and 630Δerm ΔspoZ-CD16671 (MC2351) at H12 on 70:30 agar. *, P = 0.03 by one-way ANOVA followed by Dunnett’s multiple comparisons test. (TIFF) [file pgen.1010841.s008.tiff]

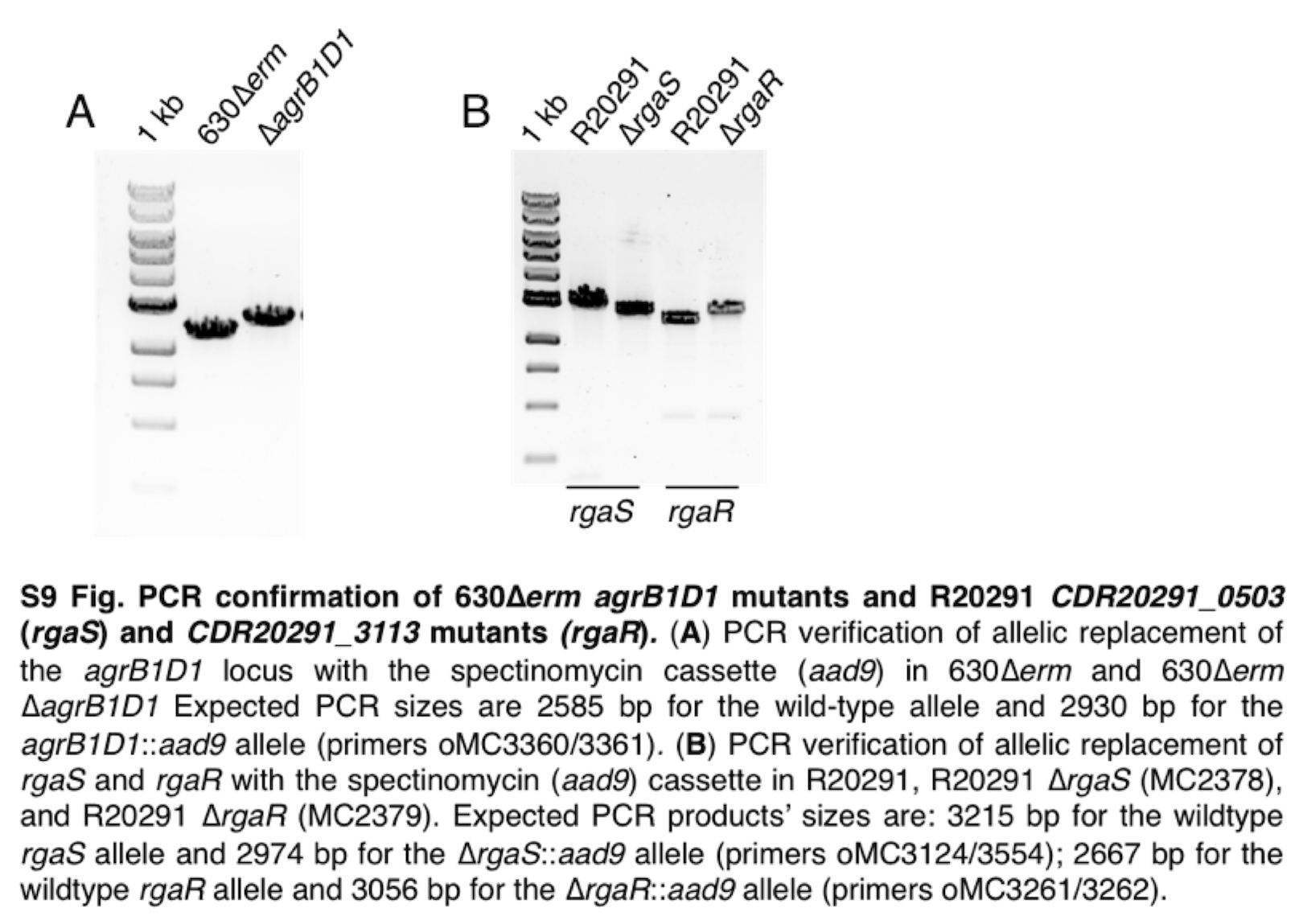

Supplement: S9 Fig — (A) PCR verification of allelic replacement of the agrB1D1 locus with the spectinomycin cassette (aad9) in 630Δerm and 630Δerm ΔagrB1D1 Expected PCR sizes are 2585 bp for the wild-type allele and 2930 bp for the agrB1D1::aad9 allele (primers oMC3360/3361). (B) PCR verification of allelic replacement of rgaS and rgaR with the spectinomycin (aad9) cassette in R20291, R20291 ΔrgaS (MC2378), and R20291 ΔrgaR (MC2379). Expected PCR products’ sizes are: 3215 bp for the wildtype rgaS allele and 2974 bp for the ΔrgaS::aad9 allele (primers oMC3124/3554); 2667 bp for the wildtype rgaR allele and 3056 bp for the ΔrgaR::aad9 allele (primers oMC3261/3262). (TIFF) [file pgen.1010841.s009.tiff]

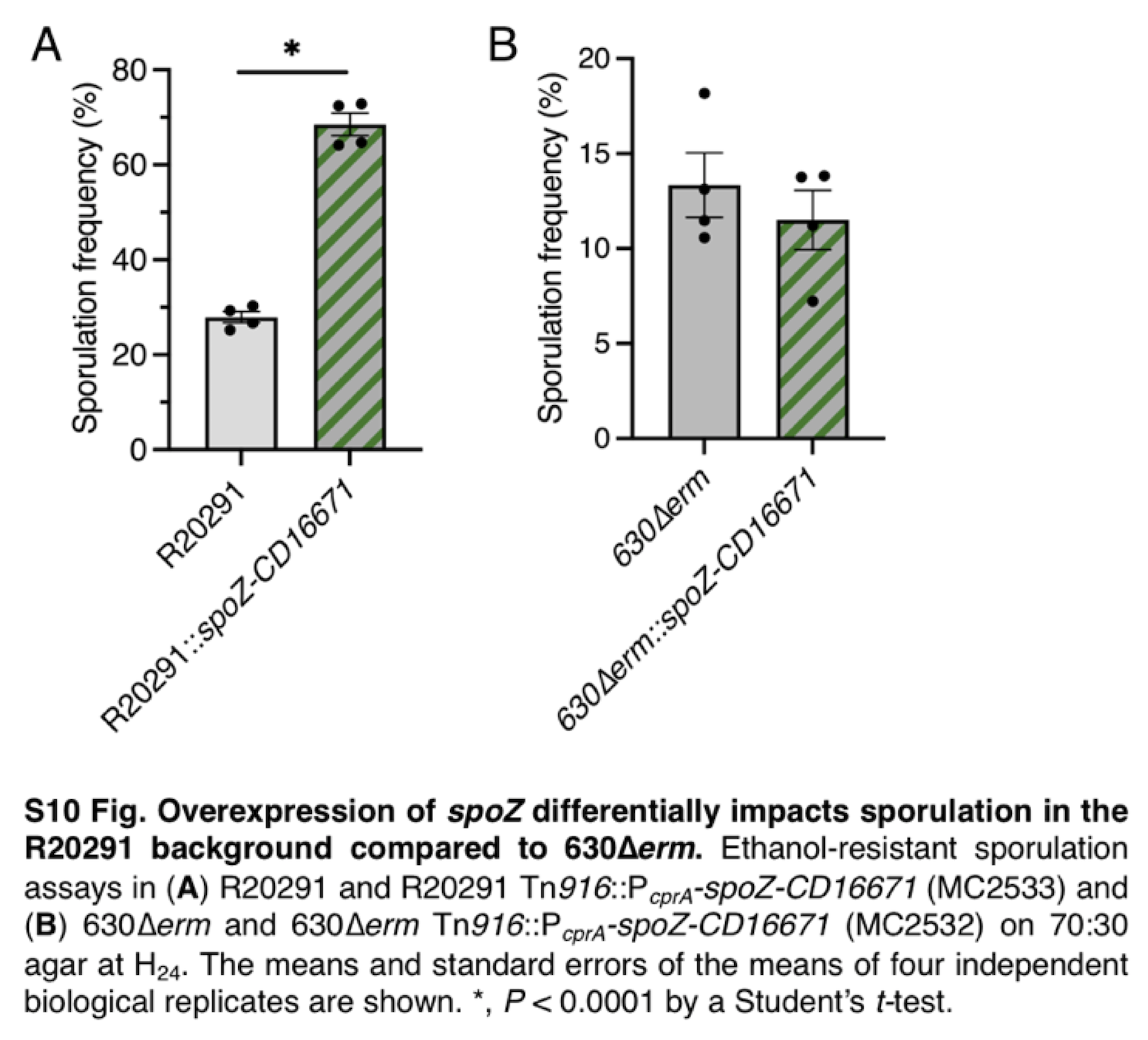

Supplement: S10 Fig — Ethanol-resistant sporulation assays in (A) R20291 and R20291 Tn916::PcprA-spoZ-CD16671 (MC2533) and (B) 630Δerm and 630Δerm Tn916::PcprA-spoZ-CD16671 (MC2532) on 70:30 agar at H24. The means and standard errors of the means of four independent biological replicates are shown. (TIFF) [file pgen.1010841.s010.tiff]
