# Supplementary material for: The RgaS-RgaR two-component system promotes Clostridioides difficile sporulation through a small RNA and the Agr1 system
Source: PLoS Genet. 2023 Oct 16;19(10):e1010841. doi: 10.1371/journal.pgen.1010841 (PMC10602386; doi:10.1371/journal.pgen.1010841)
Supplement: S1 Text. DNA cloning and vector details — (DOCX) [file pgen.1010841.s011.docx]

S1 Text - DNA cloning and vector details.

pMC1123: The 340 bp *cprA* promoter was amplified with primers oMC3060/3061 to replace the *xyl* promoter in pIA33 via Gibson assembly at the *Nhe*I and *Sal*I sites.

pMC1132: A 140 bp PCR product containing sgRNA-*rgaS* was created using primers 4084 and oMC3066 and was amplified again with primers oMC3088/3089, which contain homology to pMC1123. The resulting product was Gibson assembled into pMC1123 via the *Msc*I and *Not*I sites.

pMC1183: A 140 bp PCR product containing sgRNA-*rgaR* was created using primers 4084 and oMC3254 and was amplified again with primers oMC3088/3089, which contain homology to pMC1123. The resulting product was Gibson assembled into pMC1123 via the *Msc*I and *Not*I sites.

pMC1184: The 872 bp 5ʹ *rgaS* homology arm and 829 bp 3ʹ *rgaS* homology arm were amplified with primers oMC3222/3223 and oMC3226/3227, respectively. The 1045 bp *aad9* cassette from pMC404 was amplified using primers oMC3224/3225, and all three fragments were Gibson assembled into pMSR via the *Bam*HI and *Xho*I sites.

pMC1185: The 917 bp 5ʹ *rgaR* homology arm and 785 bp 3ʹ *rgaR* homology arm were amplified with primers oMC3255/3256 and oMC3259/3260, respectively. The 1520 bp *ermB* cassette from pJIR1457 was amplified using primers oMC3257/3258, and all three fragments were Gibson assembled into pMSR via the *Bam*HI and *Xho*I sites.

pMC1198: A 140 bp PCR product containing sgRNA-*CD0587* was created using primers 4084 and oMC3283 and was amplified again with primers oMC3088/3089, which contain homology to pMC1123. The resulting product was Gibson assembled into pMC1123 via the *Msc*I and *Not*I sites.

pMC1199: A 140 bp PCR product containing sgRNA-*CD2098* was created using primers 4084 and oMC3284 and was amplified again with primers oMC3088/3089, which contain homology to pMC1123. The resulting product was Gibson assembled into pMC1123 via the *Msc*I and *Not*I sites.

pMC1200: A 140 bp PCR product containing sgRNA-*CD15111* was created using primers 4084 and oMC3285 and was amplified again with primers oMC3088/3089, which contain homology to pMC1123. The resulting product was Gibson assembled into pMC1123 via the *Msc*I and *Not*I sites.

pMC1201: A 140 bp PCR product containing sgRNA-*spoZ* was created using primers 4084 and oMC3286 and was amplified again with primers oMC3088/3089, which contain homology to pMC1123. The resulting product was Gibson assembled into pMC1123 via the *Msc*I and *Not*I sites.

pMC1202: A 1244 bp PCR product containing *rgaR* driven by its native promoter was amplified with primers oMC3297/3298 and Gibson assembled into pSMB47 via the *Bam*HI and *Sph*I sites.

pMC1204: An 1862 bp PCR product containing *rgaS* driven by its native promoter was amplified with primers oMC3295/3296 and Gibson assembled into pSMB47 via the *Bam*HI and *Sph*I sites.

pMC1208: A site-directed *rgaS*-H256A mutant was generated using primers oMC3299/3300 and pMC1204 as the template following the manufacturer’s instructions from Agilent’s Quikchange II Site-directed mutagenesis kit.

pMC1209: A site-directed *rgaR*-D57A mutant was generated using primers oMC3301/3302 and pMC1202 as the template following the manufacturer’s instructions from Agilent’s Quikchange II Site-directed mutagenesis kit

pMC1212: The *aad9* cassette was amplified from pMC404 using primers oMC3358/3359 and Gibson assembled into pSMB47 at the *Hind*III and *Sna*BI sites to replace the *ermB* cassette.

pMC1217: A 1244 bp PCR product containing *rgaR* driven by its native promoter was amplified with primers oMC3297/3298 and Gibson assembled into pMC1212 via the *Bam*HI and *Sph*I sites.

pMC1218: A 1244 bp PCR product containing the *rgaR*-D57A allele was amplified with primers oMC3297/3298 and pMC1209 as the template and Gibson assembled into pMC1212 via the *Bam*HI and *Sph*I sites.

pMC1225: A 286 bp PCR product encompassing the *spoZ* promoter was amplified with primers oMC3459/3460 and Gibson assembled immediately upstream of *phoZ* in pMC358 via the *Eco*RI and *Bam*HI sites.

pMC1228: The 721 bp 5ʹ *spoZ-CD16671* homology arm and 753 bp 3ʹ *spoZ-CD16671* homology arm were amplified with primers oMC3447/3448 and oMC3452/3453, respectively. The 1045 bp *aad9* cassette from pMC404 was amplified using primers oMC3450/3455, and all three fragments were Gibson assembled into pMSR via the *Bam*HI and *Xho*I sites.

pMC1229: The 872 bp 5ʹ *CDR20291_0503* (*rgaS*) homology arm and 829 bp 3ʹ *CDR20291_0503* (*rgaS*) homology arm were amplified with primers oMC3463/3223 and oMC3226/3438, respectively. The 1045 bp *aad9* cassette from pMC404 was amplified using primers oMC3224/3225, and all three fragments were Gibson assembled into pMSR0 via the *Bam*HI and *Xho*I sites.

pMC1230: The 917 bp 5ʹ *CDR20291_3113* (*rgaR*) homology arm and 785 bp 3ʹ *CDR20291_3113* (*rgaR*) homology arm were amplified with primers oMC3439/3256 and oMC3440/3259, respectively. The 1045 bp *aad9* cassette from pMC404 was amplified using primers oMC3482/3483, and all three fragments were Gibson assembled into pMSR0 via the *Bam*HI and *Xho*I sites.

pMC1249: A 286 bp site-directed P*spoZ*_G-51A/G-72A_ mutant was synthesized and cloned into pMC358 by Genscript (Piscataway, NJ).

pMC1250: An allelic exchange construct containing a 1050 bp 5ʹ *CD16671* homology arm and a 753 bp 3ʹ *CD16671* homology arm flanking the 1045 bp *aad9* cassette was synthesized and cloned into pMSR by Genscript (Piscataway, NJ).

pMC1253: A 191 bp PCR product containing the open reading frame of *CD16671* was amplified with primers oMC3524/3526 and Gibson assembled into pMC211 via the *Bam*HI and *Pst*I sites.

pMC1265: A 140 bp PCR product containing sgRNA-*agrB1* was created using primers 4084 and oMC3575 and was amplified again with primers oMC3088/3089, which contain homology to pMC1123. The resulting product was Gibson assembled into pMC1123 via the *Msc*I and *Not*I sites.

pMC1271: PCR products containing the *cprA* promoter (340 bp) and the *agrB1D1* locus (1087 bp) were amplified using primers oMC2896/3593 and oMC3594/3595, respectively, and Gibson assembled into pMC1212 via the *Bam*HI and *Sph*I sites.

pMC1272: PCR products containing the *cprA* promoter (340 bp) and the s*poZ-CD16671* locus (1087 bp) were amplified using primers oMC2896/3596 and oMC3597/3598, respectively, and Gibson assembled into pMC1212 via the *Bam*HI and *Sph*I sites.
