# Supplementary material for: The RgaS-RgaR two-component system promotes Clostridioides difficile sporulation through a small RNA and the Agr1 system
Source: PLoS Genet. 2023 Oct 16;19(10):e1010841. doi: 10.1371/journal.pgen.1010841 (PMC10602386; doi:10.1371/journal.pgen.1010841)
Supplement: S2 Table. Bacterial strains and plasmids — (DOCX) [file pgen.1010841.s013.docx]

**Table S2. Bacterial strains and plasmids**

| **Plasmid or Strain** | | **Relevant genotype or features** | **Source, construction or reference** |
| --- | --- | --- | --- |
| **Strains** | |  |  |
| E. coli | |  |  |
|  | HB101 | F^-^ *mcrB mrr hsdS20*(r_B_^-^ m_B_*^-^) recA13 leuB6 ara-14* *proA2 lacY1 galK2 xyl-5 mtl-1 rpsL20* | B. Dupuy |
|  |  |  |  |
| C. difficile | |  |  |
|  | 630Δ*erm* | Erm^S^ derivative of strain 630; Ribotype 012 | Nigel Minton; [1] |
|  | R20291 | Ribotype 027 | [2] |
|  | MC282 | 630∆*erm* pMC211 | [3] |
|  | MC448 | 630∆*erm* pMC358 | [4] |
|  | MC2065 | 630∆*erm* pMC1123 | This study |
|  | MC2066 | 630∆*erm* pMC1132 | This study |
|  | MC2227 | 630∆*erm* pMC1183 | This study |
|  | MC2228 | 630∆*erm* ∆*rgaS*::*aad9* | This study |
|  | MC2229 | 630∆*erm* ∆*rgaR*::*erm* | This study |
|  | MC2236 | 630∆*erm* ∆*rgaS*::*aad9* ∆*rgaR*::*erm* | This study |
|  | MC2237 | R20291 pMC1123 | This study |
|  | MC2269 | 630∆*erm* pMC1198 | This study |
|  | MC2270 | 630∆*erm* pMC1199 | This study |
|  | MC2271 | 630∆*erm* pMC1200 | This study |
|  | MC2272 | 630∆*erm* pMC1201 | This study |
|  | MC2278 | 630∆*erm* ∆*rgaS* Tn*916*::*rgaS* | This study |
|  | MC2318 | 630∆*erm* ∆*rgaS* Tn*916*::*rgaS-*H256A | This study |
|  | MC2319 | 630∆*erm* ∆*rgaS* Tn*916*::*rgaR* | This study |
|  | MC2320 | 630∆*erm* ∆*rgaS* Tn*916*::*rgaR*-D57A | This study |
|  | MC2329 | 630∆*erm* pMC1225 | This study |
|  | MC2330 | 630∆*erm* ∆*rgaR* pMC1225 | This study |
|  | MC2351 | 630∆*erm* ∆*spoZ-CD16671*::*aad9* | This study |
|  | MC2357 | 630∆*erm* pMC1249 | This study |
|  | MC2358 | 630∆*erm* ∆*rgaR* pMC1249 | This study |
|  | MC2363 | 630∆*erm* ∆*CD16671* | This study |
|  | MC2378 | R20291 ∆*CDR20291_0503*(*rgaS*)::*aad9* | This study |
|  | MC2379 | R20291 ∆*CDR20291_3113*(*rgaR*)::*aad9* | This study |
|  | MC2424 | 630∆*erm* ∆*rgaR* Tn*916*::P*_cprA_-agrB1D1* | This study |
|  | MC2425 | 630∆*erm* ∆*rgaR* Tn*916*::P*_cprA_-spoZ-CD16671* | This study |
|  | MC2533 | R20291 Tn*916*::P*_cprA_*-*spoZ-CD16671* | This study |
|  | MC2534 | R20291 pMC1265 | This study |
|  | MC2561 | 630∆*erm* pMC1253 | This study |
|  |  |  |  |
| *B. subtilis* | |  |  |
|  | BS49 | CU2189::Tn*916* | P. Mullany |
|  |  |  |  |
| **Plasmids** | |  |  |
|  | pRK24 | Tra^+^, Mob^+^; *bla, tet* | [5] |
|  | pMSR | Pseudo-suicide plasmid used for allele exchange in *C. difficile* 630; P*tet-CD2571.1 catP* | [6] |
|  | pMSR0 | Pseudo-suicide plasmid used for allele exchange in *C. difficile* R20291; *rCd8* P*tet-CD2571.1 catP* | [6] |
|  | pSMB47 | Tn*916* integrational vector; CmR, ErmR | [7] |
|  | pJIR1457 | *ermB oriCP oriEC oriT* | [8] |
|  | pIA33 | P*_xyl_::dCas9-opt*P*_gdh_*::sgRNA-*rfp catP* | C. Ellermeier; [9] |
|  | pMC123 | *E. coli- C. difficile* shuttle vector*, bla, catP* | [10] |
|  | pMC211 | pMC123 with *cprA* promoter | [3] |
|  | pMC358 | pMC123 *phoZ* | [4] |
|  | pMC404 | pMC123 with *catP* replaced by *aad9* | [11] |
|  | pMC1123 | P*_cprA_::dCas9-opt*P*_gdh_*::sgRNA*-neg catP* | This study |
|  | pMC1132 | P*_cprA_::dCas9-opt*P*_gdh_*::sgRNA-*rgaS catP* | This study |
|  | pMC1183 | P*_cprA_::dCas9-opt*P*_gdh_*::sgRNA-*rgaR catP* | This study |
|  | pMC1184 | pMSR with *rgaS* homology regions flanking *aad9* | This study |
|  | pMC1185 | pMSR with *rgaR* homology regions flanking *ermB* | This study |
|  | pMC1198 | P*_cprA_::dCas9-opt*P*_gdh_*::sgRNA-*CD0587 catP* | This study |
|  | pMC1199 | P*_cprA_::dCas9-opt*P*_gdh_*::sgRNA-*CD2098 catP* | This study |
|  | pMC1200 | P*_cprA_::dCas9-opt*P*_gdh_*::sgRNA-*CD15111 catP* | This study |
|  | pMC1201 | P*_cprA_::dCas9-opt*P*_gdh_*::sgRNA-*spoZ catP* | This study |
|  | pMC1202 | pSMB47 with Tn*916*::*rgaR* | This study |
|  | pMC1204 | pSMB47 with Tn*916*::*rgaS* | This study |
|  | pMC1208 | pSMB47 with Tn*916*::*rgaS-*H256A | This study |
|  | pMC1209 | pSMB47 with Tn*916*::*rgaR*-D57A | This study |
|  | pMC1212 | pSMB47 with *ermB* replaced by *aad9* | This study |
|  | pMC1217 | pMC1212 with Tn*916*::*rgaR* | This study |
|  | pMC1218 | pMC1212 with Tn*916*::*rgaR*-D57A | This study |
|  | pMC1225 | pMC123 P*spoZ*::*phoZ* | This study |
|  | pMC1228 | pMSR with *spoZ-CD16671* homology regions flanking *aad9* | This study |
|  | pMC1229 | pMSR0 with *CDR20291_0503* (*rgaS*) homology regions flanking *aad9* | This study |
|  | pMC1230 | pMSR0 with *CDR20291_3113* (*rgaR*) homology regions flanking *aad9* | This study |
|  | pMC1249 | pMC123 P*spoZ_G_*_-51A/G-72A_::*phoZ* | This study |
|  | pMC1250 | pMSR with *CD16671* homology regions flanking *aad9* | This study |
|  | pMC1253 | pMC211 with *CD16671* | This study |
|  | pMC1265 | P*_cprA_::dCas9-opt*P*_gdh_*::sgRNA*-agrB1 catP* | This study |
|  | pMC1271 | pMC1212 with P*_cprA_*-*agrB1D1* | This study |
|  | pMC1272 | pMC1212 with P*_cprA_*-*spoZ-CD16671* | This study |

**REFERENCES**

1. Hussain HA, Roberts AP, Mullany P. Generation of an erythromycin-sensitive derivative of *Clostridium difficile* strain 630 (630Δ*erm*) and demonstration that the conjugative transposon Tn*916*ΔE enters the genome of this strain at multiple sites. J Med Microbiol. 2005;54: 137–141.

2. Stabler RA, He M, Dawson L, Martin M, Valiente E, Corton C, et al. Comparative genome and phenotypic analysis of *Clostridium difficile* 027 strains provides insight into the evolution of a hypervirulent bacterium. Genome Biol. 2009;10: R102. doi:10.1186/gb-2009-10-9-r102

3. Edwards AN, Nawrocki KL, McBride SM. Conserved oligopeptide permeases modulate sporulation initiation in *Clostridium difficile*. Infect Immun. 2014;82: 4276–91. doi:10.1128/IAI.02323-14

4. Edwards AN, Pascual RA, Childress KO, Nawrocki KL, Woods EC, McBride SM. An alkaline phosphatase reporter for use in *Clostridium difficile*. Anaerobe. 2015;32: 98–104.

5. Thomas CM, Smith CA. Incompatibility group P plasmids: genetics, evolution, and use in genetic manipulation. Annu Rev Microbiol. 1987;41: 77–101.

6. Peltier J, Hamiot A, Garneau JR, Boudry P, Maikova A, Hajnsdorf E, et al. Type I toxin-antitoxin systems contribute to the maintenance of mobile genetic elements in *Clostridioides difficile*. Commun Biol. 2020;3: 718. doi:10.1038/s42003-020-01448-5

7. Manganelli R, Provvedi R, Berneri C, Oggioni MR, Pozzi G. Insertion vectors for construction of recombinant conjugative transposons in *Bacillus subtilis* and *Enterococcus faecalis*. FEMS Microbiol Lett. 1998;168: 259–268.

8. Lyras D, Rood JI. Conjugative Transfer of RP4-oriTShuttle Vectors from *Escherichia coli* to *Clostridium perfringens*. Plasmid. 1998;39: 160–164.

9. Müh U, Pannullo AG, Weiss DS, Ellermeier CD. A Xylose-Inducible Expression System and a CRISPR Interference Plasmid for Targeted Knockdown of Gene Expression in *Clostridioides difficile*. J Bacteriol. 2019;201. doi:10.1128/JB.00711-18

10. McBride SM, Sonenshein AL. Identification of a genetic locus responsible for antimicrobial peptide resistance in *Clostridium difficile*. Infect Immun. 2011;79: 167–76. doi:10.1128/IAI.00731-10

11. Purcell EB, McKee RW, Courson DS, Garrett EM, McBride SM, Cheney RE, et al. A Nutrient-Regulated Cyclic Diguanylate Phosphodiesterase Controls *Clostridium difficile* Biofilm and Toxin Production during Stationary Phase. Infect Immun. 2017;85: e00347-17. doi:10.1128/IAI.00347-17
