# Supplementary material for: The RgaS-RgaR two-component system promotes Clostridioides difficile sporulation through a small RNA and the Agr1 system
Source: PLoS Genet. 2023 Oct 16;19(10):e1010841. doi: 10.1371/journal.pgen.1010841 (PMC10602386; doi:10.1371/journal.pgen.1010841)
Supplement: S3 Table. Oligonucleotides — (DOCX) [file pgen.1010841.s014.docx]

**Table S3. Oligonucleotides**

| Primer | Sequence (5ʹ🡪3ʹ)^a^ | Use/locus tag/reference |
| --- | --- | --- |
| oMC44 | 5′CTAGCTGCTCCTATGTCTCACATC | Forward primer for *rpoC* qPCR [1] |
| oMC45 | 5′CCAGTCTCTCCTGGATCAACTA | Reverse primer for *rpoC* qPCR [1] |
| oMC339 | 5′GGGCAAATATACTTCCTCCTCCAT | Forward primer for *sigE* qPCR [2] |
| oMC340 | 5′TGACTTTACACTTTCATCTGTTTCTAGC | Reverse primer for *sigE* qPCR [2] |
| oMC427 | 5′GTGGTGTTAATACATCAGAACTTCC | Forward primer for *sigG* qPCR [2] |
| oMC428 | 5′CAAACTGTTGTCTGGCTTCTTC | Reverse primer for *sigG* qPCR [2] |
| oMC547 | 5′TGGATAGGTGGAGAAGTCAGT | Forward primer for *tcdA* qPCR [2] |
| oMC548 | 5′GCTGTAATGCTTCAGTGGTAGA | Reverse primer for *tcdA* qPCR [2] |
| oMC2896 | 5′GACCACACCCGTCCTGTGGATCCGACATGGAAGTAGAAGTTAAGGAAAAAGAT | Forward primer for P*cprA* to Gibson assemble into pSMB47/pMC1212 |
| oMC3060 | 5′CTTAAGGGCGAATTCTGCATCAAGCTAGCGACATGGAAGTAGAAGTTAAGG | Forward primer for P*cprA* to Gibson assemble into pIA33 |
| oMC3061 | 5′CCTATTGCTAATCCTATACTATATTTTTTATCCATGCGTCGACCATCCTTTCTTTTGTCAAACTT | Reverse primer for P*cprA* to Gibson assemble into pIA33 |
| oMC3066 | 5′AATTAAACTGTAAATGGCCATAAATGACATGAGTAGTATGGTTTTAGAGCTAGAAATAGC | Forward primer for sgRNA-*rgaS* amplification |
| oMC3074 | 5′TGCAGGTAGAAATTCAGAGTAATG | Forward primer for *rgaS* qPCR |
| oMC3075 | 5′TCAACAGCTTCACAACAACTAA | Reverse primer for *rgaS* qPCR |
| oMC3088 | 5′TTGCAATAAAGTGTGCTATAATTAAACTGTAAATGGCCA | Forward primer to Gibson assemble CRISPRi sgRNAs into pMC1123 |
| oMC3089 | 5′CCTTTTTCTATTTAAAGTTTTATTAAAACTTATAGGATCCGCGGCCGC | Reverse primer to Gibson assemble CRISPRi sgRNAs into pMC1123 |
| oMC3123 | 5′CCTTCTAACTTAGATGTAAGAGTTATAGC | Forward primer to screen *rgaS* mutants |
| oMC3124 | 5′GGTTCAGATGTTAAACCAGAGC | Reverse primer to screen *rgaS/CDR20291_0503* mutants |
| oMC3222 | 5′CTCTACAGCAAAATAGATTGTAGTTCTTCGGATCCGAGGTGCTTATATCATGAATAAAAGTAGAAAT | Forward primer to amplify *rgaS* 5′ flanking region and Gibson assemble into pMSR |
| oMC3223 | 5′GTCTTGCCAGTCACGTTACGTCGACAATACTTTCTCCTAACGTATTACTAAATTATTTTTTTA | Reverse primer to amplify *rgaS* 5′ flanking region with homology to *aad9* |
| oMC3224 | 5′TAAAAAAATAATTTAGTAATACGTTAGGAGAAAGTATTGTCGACGTAACGTGACTGGCAAGAC | Forward primer to amplify *aad9* cassette with homology to *rgaS* 5′ flanking region |
| oMC3225 | 5′ATTAATTCTTATATATTAAATTTCTACACATCAAATTTATATACATGTCGACACCCAAAATTGAAAAAAGTGTTTCC | Reverse primer to amplify *aad9* cassette with homology to *rgaS* 3′ flanking region |
| oMC3226 | 5′GGAAACACTTTTTTCAATTTTGGGTGTCGACATGTATATAAATTTGATGTGTAGAAATTTAATATATAAGAATTAAT | Forward primer to amplify *rgaS* 3′ flanking region with homology to *aad9* |
| oMC3227 | 5′CTTGCATGTCTGCAGGCCTCGAGGGCTGTTCTCGAGACGTTATAAG | Reverse primer to amplify *rgaS* 3′ flanking region and Gibson assemble into pMSR |
| oMC3254 | 5′AATTAAACTGTAAATGGCCAAGATATCAATGGAATTGAGGGTTTTAGAGCTAGAAATAGC | Forward primer for sgRNA-*rgaR* amplification |
| oMC3255 | 5′CTCTACAGCAAAATAGATTGTAGTTCTTCGGATCCGGCTGAAGAAGAAGATATGATGAACTTA | Forward primer to amplify *rgaR* 5′ flanking region and Gibson assemble into pMSR |
| oMC3256 | 5′ATAATCTCATGACCAAAATCCCTTAACGGTCGACAATATCACCTCCAAATCTAAAATATCATTTAGTTTAC | Reverse primer to amplify *rgaR* 5′ flanking region with homology to *ermB* |
| oMC3257 | 5′GTAAACTAAATGATATTTTAGATTTGGAGGTGATATTGTCGACCGTTAAGGGATTTTGGTCATGAGATTAT | Forward primer to amplify *ermB* cassette with homology to *rgaR* 5′ flanking region |
| oMC3258 | 5′TTCAATCAATAGCCCTGTGATGACGTCGACCTTGGAAGCTGTCAGTAGTATACCT | Reverse primer to amplify *ermB* cassette with homology to *rgaR* 3′ flanking region |
| oMC3259 | 5′AGGTATACTACTGACAGCTTCCAAGGTCGACGTCATCACAGGGCTATTGATTGAA | Forward primer to amplify *rgaR* 3′ flanking region with homology to *ermB* |
| oMC3260 | 5′CTTGCATGTCTGCAGGCCTCGAGGGAGATTATCTTTTTATTAGACAATCCCC | Reverse primer to amplify *rgaR* 3′ flanking region and Gibson assemble into pMSR |
| oMC3261 | 5′GGAAATATATACTCATCTTCCTACTGTT | Forward primer to screen *rgaR/CDR20291_3113* mutants |
| oMC3262 | 5′CTTGCTAAAAACATAAAATAAGTACACCAT | Reverse primer to screen *rgaR/CDR20291_3113* mutants |
| oMC3271 | 5′TGAAAGATATCAATGGAATTGAGGTG | Forward primer for *rgaR* qPCR |
| oMC3272 | 5′AAATATCTATAAGCACGAACCTCATAAC | Reverse primer for *rgaR* qPCR |
| oMC3283 | 5ʹAATTAAACTGTAAATGGCCAACTTGAGTAGAAACAATAAGGTTTTAGAGCTAGAAATAGC | Forward primer for sgRNA-*CD0587* amplification |
| oMC3284 | 5ʹAATTAAACTGTAAATGGCCAGAATGTTTAGTCAGCTTTGAGTTTTAGAGCTAGAAATAGC | Forward primer for sgRNA-*CD2098* amplification |
| oMC3285 | 5ʹAATTAAACTGTAAATGGCCATCATTCATAGAAACTCAAATGTTTTAGAGCTAGAAATAGC | Forward primer for sgRNA-*CD15111* amplification |
| oMC3286 | 5ʹAATTAAACTGTAAATGGCCAGAGAATGTATAAAATAGTAAGTTTTAGAGCTAGAAATAGC | Forward primer for sgRNA-*spoZ* amplification |
| oMC3289 | 5ʹTTCAGAGCTGTCGAAGAAGAAG | Forward primer for *CD0587* qPCR |
| oMC3290 | 5ʹCTTGAGTAGAAACAATAAGCGGTTT | Reverse primer for *CD0587* qPCR |
| oMC3291 | 5ʹGCTTTGAGGGTCGTTCAATAAG | Forward primer for *CD2098* qPCR |
| oMC3292 | 5ʹCCAATTTAGAAATTTCCTCTTCTGC | Reverse primer for *CD2098* qPCR |
| oMC3295 | 5ʹGACCACACCCGTCCTGTGGATCCATTAATTCTTATATATTAAATTTCTACACATCAAATTTATATACAT | Forward primer to amplify *rgaS* to Gibson assemble into pSMB47 |
| oMC3296 | 5ʹCCGCCGCAAGGAATGGTGCATGCGCTTTTGTTGTAAGAATCATTTTATCTAATGA | Reverse primer to amplify *rgaS* and Gibson assemble into pSMB47 |
| oMC3297 | 5ʹGACCACACCCGTCCTGTGGATCCCTGTTGAGGAAAGATTAACAAATTTAGAATC | Forward primer to amplify *rgaR* and Gibson assemble into pSMB47 |
| oMC3298 | 5ʹCCGCCGCAAGGAATGGTGCATGCTTCAATCAATAGCCCTGTGATGAC | Reverse primer to amplify *rgaR* and Gibson assemble into pSMB47 |
| oMC3299 | 5ʹAATCATAGCATTTATACACAATATGTGATTTTTCATATCTGCACTTAGTAATCTAGTTTTTAAATATTTCTTTTCTATATC | Forward primer for site-directed mutagenesis of RgaS-H256A (Agilent Quikchange) |
| oMC3300 | 5ʹGATATAGAAAAGAAATATTTAAAAACTAGATTACTAAGTGCAGATATGAAAAATCACATATTGTGTATAAATGCTATGATT | Reverse primer for site-directed mutagenesis of RgaS-H256A (Agilent Quikchange) |
| oMC3301 | 5ʹCTCAATTCCATTGATATCTTTCATTTGGATTGCTAAAAATATAATATCAATTTTTTCTGGGTATTTGG | Forward primer for site-directed mutagenesis of RgaR-D57A (Agilent Quikchange) |
| oMC3302 | 5ʹCCAAATACCCAGAAAAAATTGATATTATATTTTTAGCAATCCAAATGAAAGATATCAATGGAATTGAG | Reverse primer for site-directed mutagenesis of RgaR-D57A (Agilent Quikchange) |
| oMC3303 | 5ʹTGATGAATGTGTTCATATAATGACTGC | Forward primer for *CD15111* qPCR |
| oMC3304 | 5ʹCATAACTCTACAACATCTTCTCCATATTT | Reverse primer for *CD15111* qPCR |
| oMC3305 | 5ʹCCATGTTTAGTGGATTGCAGAATA | Forward primer for *spoZ* qPCR |
| oMC3306 | 5ʹGGAGTACATCTTTACTTTCCCATTATAG | Reverse primer for *spoZ* qPCR |
| oMC3353 | 5ʹTGGAGAAATAGACAAATGTACACATATACTAAC | Forward primer for *CD16671* qPCR |
| oMC3354 | 5ʹGGTTTCAACTACCGTTTCTCCATATTC | Reverse primer for *CD16671* qPCR |
| oMC3358 | 5ʹGCAATTTAACTGTGATAAACTACCGCATTAAAGCTTGTAACGTGACTGGCAAGAC | Forward primer to amplify *aad9* cassette and Gibson assemble into pSMB47 |
| oMC3359 | 5ʹCTCGATTGACCCATTTTGAAACAAAGTACGTAACCCAAAATTGAAAAAAGTGTTTCC | Reverse primer to amplify *aad9* cassette and Gibson assemble into pSMB47 |
| oMC3438 | 5ʹGCTTGCATGTCTGCAGGCCTCGAGGGCTGTTCTCGAGACGTTATAAG | Reverse primer to amplify *CDR20291_0503* (*rgaS*) 3ʹ flanking region and Gibson assemble into pMSR0 |
| oMC3439 | 5ʹGGTGTCCATTGATTTCTTTCAGTTTCGGATCCGGCTGAAGAAGAAGATATGATGAACTTA | Forward primer to amplify *CDR20291_3113* (*rgaR*) 5′ flanking region and Gibson assemble into pMSR0 |
| oMC3440 | 5ʹGCTTGCATGTCTGCAGGCCTCGAGGGAGATTATCTTTTTATTAGACAATCCCC | Reverse primer to amplify *CDR20291_3113* (*rgaR*) 3′ flanking region and Gibson assemble into pMSR0 |
| oMC3447 | 5ʹCTCTACAGCAAAATAGATTGTAGTTCTTCGGATCCGGGTATTTTCTAATACTTTCACATCCATATTC | Forward primer to amplify *spoZ-CD16671* 5′ flanking region and Gibson assemble into pMSR |
| oMC3448 | 5ʹGTCTTGCCAGTCACGTTACGTCGACCACCTATAAAATAACACCAATATTTAGAATATTCAAT | Reverse primer to amplify *spoZ-CD16671* 5′ flanking region with homology to *aad9* |
| oMC3450 | 5ʹATTGAATATTCTAAATATTGGTGTTATTTTATAGGTGGTCGACGTAACGTGACTGGCAAGAC | Forward primer to amplify *aad9* cassette with homology to *spoZ-CD16671* 5′ flanking region |
| oMC3452 | 5ʹCTTGCATGTCTGCAGGCCTCGAGCCTACGTCGATACCACTTCTC | Reverse primer to amplify *spoZ-CD16671* 3′ flanking region and Gibson assemble into pMSR |
| oMC3453 | 5ʹGGAAACACTTTTTTCAATTTTGGGTGTCGACATTATCCATCCCCCTAAAAAATTTAGTAAAAATAA | Forward primer to amplify *spoZ-CD16671* 3′ flanking region with homology to *aad9* |
| oMC3455 | 5ʹTTATTTTTACTAAATTTTTTAGGGGGATGGATAATGTCGACACCCAAAATTGAAAAAAGTGTTTCC | Reverse primer to amplify *aad9* cassette with homology to *spoZ-CD16671* 3′ flanking region |
| oMC3457 | 5ʹGTTAAATTTTCTTGCAAAATCTCAAAAAATACC | Forward primer to screen *spoZ-CD16671* mutants |
| oMC3458 | 5ʹCCTTATATTCATTCACACTTAGATTATCAAG | Reverse primer to screen *spoZ-CD16671* mutants |
| oMC3459 | 5ʹCACGACGTTGTAAAACGACGGCCAGTATGAGAATTCGTGGATAAAAATATTGGGAGTTTAATTTTTGTG | Forward primer to amplify P*spoZ* and Gibson assemble into pMC358 |
| oMC3460 | 5ʹTTCCTCCTTCATATCTACCCATACATTGACGGATCCCACCTATAAAATAACACCAATATTTAGAATATTCAAT | Reverse primer to amplify P*spoZ* and Gibson assemble into pMC358 |
| oMC3463 | 5ʹGGTGTCCATTGATTTCTTTCAGTTTCGGATCCGTCTCATTAACTCCTGTAAAGCTACTG | Forward primer to amplify *CDR20291_0503* (*rgaS*) 5ʹ flanking region and Gibson assemble into pMSR0 |
| oMC3482 | 5ʹGTAAACTAAATGATATTTTAGATTTGGAGGTGATATTGTCGACGTAACGTGACTGGCAAGAC | Forward primer to amplify *aad9* cassette with homology to 5ʹ *CDR20291_3113* (*rgaR*) flanking region |
| oMC3483 | 5ʹTTCAATCAATAGCCCTGTGATGACgtcgacACCCAAAATTGAAAAAAGTGTTTCC | Reverse primer to amplify *aad9* cassette with homology to 3ʹ *CDR20291_3113* (*rgaR*) flanking region |
| oMC3514 | 5ʹATGTTTATTGGAGAAATAGACAAATGTAC | Reverse primer for *CD16671* amplification |
| oMC3524 | 5ʹATAAGTTTGACAAAAGAAAGGATGAAAATTGGATCCGGGGGATGGATAATATGTTTATTGG | Forward primer for *CD16671* amplification to Gibson assemble into pMC211 |
| oMC3526 | 5ʹTATGACCATGATTACGCCAAGCTGCAGTTAATTGTATCTACTTACTAATAATAAGACTTGATG | Reverse primer for *CD16671* amplification to Gibson assemble into pMC211 |
| oMC3554 | 5ʹGGTAGAGAATATGGTTCCTTAACAC | Forward primer to screen *CDR20291_0503* mutants |
| oMC3556 | 5ʹGACCATGATTACGCCAAGCTTGCATGCGAACCTACTGTCCTTATTATGGAAAG | Reverse primer to amplify *CD16671* promoter region |
| oMC3575 | 5ʹAATTAAACTGTAAATGGCCAATAACCACCATGCTGTTTATGTTTTAGAGCTAGAAATAGC | Forward primer for sgRNA-*agrB1* amplification |
| oMC3593 | 5ʹCAGCATATCTTTTAAACATTTTTACCTCCAAAATTTTCATCCTTTCTTTTGTCAAACTTATTTAC | Reverse primer to amplify *cprA* promoter with homology to *agrB1D1* |
| oMC3594 | 5ʹGTAAATAAGTTTGACAAAAGAAAGGATGAAAATTTTGGAGGTAAAAATGTTTAAAAGATATGCTG | Forward primer to amplify *agrB1D1* with homology to the *cprA* promoter |
| oMC3595 | 5ʹCCGCCGCAAGGAATGGTGCATGCCCCTTCATTATATCATAAGTTTACATAAAATACATAC | Reverse primer to amplify *agrB1D1* and Gibson assemble into pSMB47/pMC1212 |
| oMC3596 | 5ʹATCCCCCTAATATGTTTAAACCACCAATTTTCATCCTTTCTTTTGTCAAACTTATTTAC | Reverse primer to amplify *cprA* promoter with homology to *spoZ-CD16671* |
| oMC3597 | 5ʹGTAAATAAGTTTGACAAAAGAAAGGATGAAAATTGGTGGTTTAAACATATTAGGGGGAT | Forward primer to amplify *spoZ-CD16671* with homology to the *cprA* promoter |
| oMC3598 | 5ʹCCGCCGCAAGGAATGGTGCATGCGAATATTTTCTATTCTCAAACAAGCATGTTG | Reverse primer to amplify *spoZ-CD16671* and Gibson assemble into pMC1212 |
| oMC3605 | 5ʹGCTTATATTAGCAGTTCGTATGATTATTGT | Forward primer for *CD19903* qPCR |
| oMC3606 | 5ʹACTTACCAATACCATTACTTGGTGTAAA | Reverse primer for *CD19903* qPCR |
| oMC3901 | 5ʹCATATTCTGCAATCCACTAAACATGGCTCT/iAzideN/A | irNorthern DNA probe for *spoZ* |
| 4084 | 5′AACTTATAGGATCCGCGGCCGCTAGTCAGACATCATGCTGATCTAGA | Reverse primer for sgRNA amplification [3] |

^a^Restriction sites used for cloning and CRISPRi sgRNA target sequences are underlined.

**REFERENCES**

1. McBride SM, Sonenshein AL. Identification of a genetic locus responsible for antimicrobial peptide resistance in *Clostridium difficile*. Infect Immun. 2011;79: 167–76. doi:10.1128/IAI.00731-10

2. Edwards AN, Nawrocki KL, McBride SM. Conserved oligopeptide permeases modulate sporulation initiation in *Clostridium difficile*. Infect Immun. 2014;82: 4276–91. doi:10.1128/IAI.02323-14

3. Müh U, Pannullo AG, Weiss DS, Ellermeier CD. A Xylose-Inducible Expression System and a CRISPR Interference Plasmid for Targeted Knockdown of Gene Expression in *Clostridioides difficile*. J Bacteriol. 2019;201. doi:10.1128/JB.00711-18
