# Supplementary material for: The RgaS-RgaR two-component system promotes Clostridioides difficile sporulation through a small RNA and the Agr1 system
Source: PLoS Genet. 2023 Oct 16;19(10):e1010841. doi: 10.1371/journal.pgen.1010841 (PMC10602386; doi:10.1371/journal.pgen.1010841)
Supplement: Attachment — Submitted filename: Comments-PGENETICS-D-23-00713.docx [file pgen.1010841.s015.docx]

**Comments on PGENETICS-D-23-00713:**

In the present manuscript, the authors identify and functionally characterize a conserved Agr-like two component system that promotes sporulation through multiple distinct mechanisms. Of note, this Agr system differs in an important regulatory aspect from other systems in that it does not function in an autoregulatory fashion. Importantly, the present manuscript contributes significant novel insights into the molecular mechanism that lead to Spo0A phosphorylation, a critical step in sporulation initiation.

The data are very well explained and presented in an intuitive and comprehensible way. The data looks robust and reproducible, and convincingly establish RgaSR as a novel TCS that regulates the sporulation pathway. However, to improve the mechanistic understanding of this intriguing regulatory gene circuit, there are several points of the study that need to be addressed, partly with more experiments:

**Major comments:**

*Regarding the TCS:*

- Please, elaborate a bit more on which other genes (beyond sporulation sigma factor regulons) are differentially regulated in the *rgaR* knockout. With respect to direct RgaR targets, the authors limit the analysis on previously identified target genes. But analysis of the promoter regions of all differentially expressed genes in the present RNA-seq dataset might result in a more refined promotor motif for rgaR and guide identification of additional targets of the TC systems (or add confidence to the existing regulon).
- An important question that remains open, and that is neither addressed in the discussion is: Which signal activates the TC system? I acknowledge that this is not a trivial question but a computational promoter analysis and an expression analysis of *rgaRS* might at least provide a first glimpse whether the system is constitutively expressed or only under distinct conditions/growth phases.

*Regarding SrsR:*

- The sRNA gene overlaps with CD16671, please adjust this in figures 4, 8 and S5
- Please, also add the predicted secondary structure for SrsR to Figure 4 so that the reader can better appreciate whether it has the classic structural features (such as 3’ terminator stem) of Hfq-dependent sRNAs.
- I appreciate that identification of direct SrsR targets might be beyond the scope of the manuscript. However, as the authors state in the discussion, an existing RIL-seq dataset has already revealed a list of interacting mRNAs. Integrating these data with *in silico* target prediction might narrow down potential targets and allow the authors a less speculative discussion how/why SrsR impacts sporulation (or even to test by deleting target candidates).
- The authors discuss the contradicting results obtained by various SrsR experiments. However, these contradicting results could be more clearly mentioned in the results section, e.g.
  - In Fig 4A & 5A, if RgaS and RgaR influence sporulation frequency through the SrsR sRNA, knock-down or deletion of the sRNA should result in a decrease of sporulation frequency, similar to the RgaS/R deletion, not an increase
  - In Fig 7D, overexpression of the srsR sRNA also results in an increase in sporulation frequencies. Based on the previous results from Δerm the opposite would be expected (4A/5A - increased sporulation in deletion/knock down and 6C - decreased sporulation in overexpression). This is not addressed in the results section.
  - the authors speculate that the contradicting results might be due to differences in sRNA levels -> is it possible to to test this hypothesis by combining overexpression and gradual/partial knock-down and look at sporulation frequencies in these strains (supplemented with NB validation of sRNA expression)?
- Finally, I would like to suggest renaming the sRNA, since it is not the only sRNA regulating sporulation in *C. difficile*.

*Regarding CD16671* (and the lack of a phenotype)*:*

- The antisense transcription of SrsR might lead to translational inhibition or degradation of CD16671. Accordingly, the sRNA knockout could lead to increased CD16671 transcript levels. Perhaps, it would be worth testing a strain that overexpresses CD16671 (instead of a *CD16671* deletion) for a potential sporulation phenotype.
- CD16671 has homologs in the C. difficile genome. Could a redundant function be the reason the authors don’t see an effect on sporulation in the CD16671 KO mutant?

**Minor comments:**

- Line 232: promote needs an s ->… promotes sporulation …
- Sporulation assay is done by treating with 28.5% EtOH for 15 min. This is a rather low EtOH concentration, is this generally enough to kill the Δerm and the R20291 vegetative cells?
- Figure 1:
- Why does the CRISPRi knockdown work better than the complete knockout (Fig2A) in inhibiting sporulation?
- The CRISPRi promoter seems to be leaky, since there is a clear decrease of sporulation frequency in the uninduced control – if possible, show expression of the sgRNA with a northern blot uninduced and induced?
- In line, it might be better to compare induced to noninduced for the significance Test.
- Why was the standard deviation shown in A, but the standard error in C? They should be consistent with this (across the paper) for better comparability.
- Figure 2C:
- Why is there such a huge overexpression when inserting the complementation? Since the native promoter is included, this should not be the case. Are there multiple insertions or insertions into other genes affecting sporulation [I am not familiar with the method]?
- Figure description: (C) should be bold; Tukey’s multiple comparison test for Figure C instead of B?
- It might be worth verifying srsR expression via NB in a WT compared to a rgaS rgaR mutant to see, if the TC system is the only transcriptional activator of the sRNA in the tested conditions
- Table 1: What is the cutoff for up/down regulated genes?
- Figure 5B is neither mentioned nor discussed in the text.
- Figure 6B: Please correct the figure legend. According to the description the graph shows gene levels in wt, *agrB1D1* deletion, and complementation, but this isn’t shown in the figure.
- Figure 7B, line 404: There is no reference in the text
- Figure 7D: Include a bar graph with statistical Tests for sporulation in R20291 *srsR* overexpression
