# Supplementary material for: The RgaS-RgaR two-component system promotes Clostridioides difficile sporulation through a small RNA and the Agr1 system
Source: PLoS Genet. 2023 Oct 16;19(10):e1010841. doi: 10.1371/journal.pgen.1010841 (PMC10602386; doi:10.1371/journal.pgen.1010841)
Supplement: Attachment — Submitted filename: Response to Reviewers.docx [file pgen.1010841.s016.docx]

We sincerely appreciate the thorough, careful assessments provided by the editor and reviewers. Your thoughtful suggestions and comments along with the additional experiments recommended have significantly strengthened and clarified our manuscript. We are particularly grateful for the suggestion to overexpress *CD16671* as this data provided clear explanations for the conflicting sporulation phenotypes we observed. We believe our manuscript is now suitable for publication in *PLoS Genetics*.

Editor’s comments:

The Reviewers were in agreement that your discovery of a new two-component system, RgaS/RgaR in Clostridioides difficile that modulates sporulation levels through an Agr quorum-sensing system and a novel small regulatory RNA, SrsR, reveals an additional layer to the regulation of this important developmental process. While they found the manuscript to be well written and the experiments rigorously conducted, they were in agreement that additional mechanistic insight into how RgaS/RgaR promote sporulation would increase the impact of the current study. Reviewer 3 would like additional data addressing whether RgaR/RgaS impact Spo0A activity/activation. Reviewer 2 suggested some in silico analyses to potentially gain insight into how SrsR promotes sporulation (they also suggest renaming SrsR, since two small regulatory RNAs were recently shown to alter Spo0A levels and thus regulate sporulation) and would like clarification on the position of the sRNA gene with CD16671. Conducting some northern blots or RNAse protection assays to gain insight into the nature of the specific transcripts associated with srsR and CD16671 might help refine the authors’ model for how SrsR modulates sporulation. Related to the authors’ working model, SrsR appears to antagonize the levels of CD16671 transcript, so Reviewer 2 suggests over-expressing CD16671 to recapitulate the reduced sporulation mutant of rgaR/rgaS mutants.

If the authors’ data support a role for RgaS/RgaR in regulating sporulation primarily at later stages, the authors may want to consider focusing a revised manuscript more on RgaS/RgaR’s regulation of SrsR function rather than on the Agr system. This may help the reader follow the complex regulatory picture uncovered by the authors more clearly. Using a more precise nomenclature when referring to mutant strains would also improve readability, since mutants are often shown in figures without a ∆ symbol when they are knock-outs, or only a part of the mutation is referred to (e.g mutations that impact both srsR-CD16671 should be described as such rather than sometimes as an srsR deletion).

Reviewer #1: Review has been uploaded as an attachment

Comments on PGENETICS-D-23-00713:

In the present manuscript, the authors identify and functionally characterize a conserved Agr-like two component system that promotes sporulation through multiple distinct mechanisms. Of note, this Agr system differs in an important regulatory aspect from other systems in that it does not function in an autoregulatory fashion. Importantly, the present manuscript contributes significant novel insights into the molecular mechanism that lead to Spo0A phosphorylation, a critical step in sporulation initiation.

The data are very well explained and presented in an intuitive and comprehensible way. The data looks robust and reproducible, and convincingly establish RgaSR as a novel TCS that regulates the sporulation pathway. However, to improve the mechanistic understanding of this intriguing regulatory gene circuit, there are several points of the study that need to be addressed, partly with more experiments:

Major comments:

Regarding the TCS:

· Please, elaborate a bit more on which other genes (beyond sporulation sigma factor regulons) are differentially regulated in the rgaR knockout. With respect to direct RgaR targets, the authors limit the analysis on previously identified target genes. But analysis of the promoter regions of all differentially expressed genes in the present RNA-seq dataset might result in a more refined promotor motif for rgaR and guide identification of additional targets of the TC systems (or add confidence to the existing regulon).

All of the genes that were downregulated in the *rgaR* mutant were either previously identified direct targets (O’Connor *Mol Microbiol* 2006) or sporulation-specific genes that are represented in Table 1 (belonging in the Spo0A-dependent or sporulation-specific sigma factor regulons). We have added a line describing this significance of these findings in the context of the RgaR regulon (lines 267-270). Several of the upregulated genes are Spo0A-dependent genes or other genes encoding early sporulation factors, which is not surprising; a late-stage sporulation mutant will accumulate early-stage sporulation transcripts because the cells cannot progress to the next stage (Fimlaid *et al*. 2013 *PLoS Genet* PMID: 23950727). None of the upregulated genes contain a recognizable RgaR consensus sequence, suggesting that these genes are indirectly impacted by the absence of RgaR.

To further assess whether there were additional unidentified genes directly regulated by RgaR, we performed *in silico* MEME analyses using the *C. perfringens* VirR and RgaR consensus sites (O’Connor *et al*. *Mol Microbiol* 2006) on both the *C. difficile* 630∆*erm* and R20291 genomes. No additional occurrences of this direct repeat motif were found in either genome. Two additional matched sequences were discovered in both the 630∆*erm* and R20291 genomes in the promoter regions of *CD630_11250* and *CD630_19300* (corresponding to *CDR20291_0957* and *CDR20291_1853*). However, each of the consensus sites contained a single nucleotide substitution in the critical “G” fourth nucleotide position in one of the two direct repeats (i.e. CCACTT in the first direct repeat of *CD630_11250* and CCATTT). The RNA-seq data reveal that there are no significant changes in CD630_11250 or *CD630_19300* transcript levels (**Table S1**), although *CD630_19300* is a SigE-dependent gene (Fimlaid *et al.* 2013 *PLoS Genet* PMID: 23950727). Further, mutation of the same critical “G” nucleotide in each of the direct repeats in the promoter region of *spoZ* abrogate RgaR-dependent regulation (**Fig. 4B**). These data confirm that RgaR has a strict preference for binding sites and allow us to conclude that there are likely no other genes in the *C. difficile* 630 and R20291 RgaS/RgaR regulons.

· An important question that remains open, and that is neither addressed in the discussion is: Which signal activates the TC system? I acknowledge that this is not a trivial question but a computational promoter analysis and an expression analysis of rgaRS might at least provide a first glimpse whether the system is constitutively expressed or only under distinct conditions/growth phases.

We agree that identifying the signal that activates RgaS is an important and significant research focus. *rgaR* and *rgaS* are orphans and are encoded as single gene operons. Our preliminary data indicate that *rgaS*, *rgaR*, and all of the RgaR-dependent genes are constitutively expressed during growth on sporulation agar (data not shown). These data suggest that the RgaSR TCS is potentially a) not further activated in the conditions tested or b) constitutively active in these conditions. Additional studies in a variety of media will need to be performed determine the optimal conditions in which to study RgaSR activation and are beyond the scope of the current study.

Regarding SrsR:

· The sRNA gene overlaps with CD16671, please adjust this in figures 4, 8 and S5

Thank you for pointing this out. We have now modified this figure to reflect the results of our northern blots and PCR analyses of cDNA.

· Please, also add the predicted secondary structure for SrsR to Figure 4 so that the reader can better appreciate whether it has the classic structural features (such as 3’ terminator stem) of Hfq-dependent sRNAs.

SpoZ is a large regulatory sRNA (>500 nt). We utilized both UNAFold (mfold) and RNAStructure to predict a secondary structure for the 528 nt SpoZ transcript (length is based off of transcription termination mapping by Fuchs et al 2021 *PNAS* which is a little smaller than the length predicted by our irNorthern results (~560 nt), but provides nucleotide-level resolution). Both algorithms generated virtually identical structures. Because of the size of SpoZ and the inability to verify any of these predicted structural features in our current study, we’ve included the lowest free energy predicted secondary structure from UNAFold (mfold) as **Fig. S6C**.

· I appreciate that identification of direct SrsR targets might be beyond the scope of the manuscript. However, as the authors state in the discussion, an existing RIL-seq dataset has already revealed a list of interacting mRNAs. Integrating these data with in silico target prediction might narrow down potential targets and allow the authors a less speculative discussion how/why SrsR impacts sporulation (or even to test by deleting target candidates).

We have added in the RIL-seq data from the Fuchs 2023 study (Discussion, lines 579-587). Briefly, there are three identified targets: two mRNA transcripts and a newly identified sRNA (CDIF630_nc165). Determining whether the RgaSR TCS and SpoZ impact transcript levels and stability of these direct SpoZ targets are a focus of our immediate future studies.

· The authors discuss the contradicting results obtained by various SrsR experiments. However, these contradicting results could be more clearly mentioned in the results section, e.g.

o In Fig 4A & 5A, if RgaS and RgaR influence sporulation frequency through the SrsR sRNA, knock-down or deletion of the sRNA should result in a decrease of sporulation frequency, similar to the RgaS/R deletion, not an increase

We have added additional rationale to this paragraph (lines 293-297) to more directly address these contradictions.

o In Fig 7D, overexpression of the srsR sRNA also results in an increase in sporulation frequencies. Based on the previous results from Δerm the opposite would be expected (4A/5A - increased sporulation in deletion/knock down and 6C - decreased sporulation in overexpression). This is not addressed in the results section.

Thank for you pointing this out! We did need to more clearly place these R20291 results in context. We re-evaluated our data and now include data for a 630∆*erm* strain that expresses the Tn916::P*cprA-spoZ-CD16671* construct (**Fig. 7E; Fig. S10B**). Interestingly, of the three direct SpoZ targets identified in the RIL-seq data, only one of these, CDIF630_02260, is present in the R20291 genome. Further, there may be additional SpoZ targets in R20291 that are not encoded in the 630 background. The potential differences in the SpoZ regulon between R20291 and 630∆*erm* may explain the different sporulation phenotypes observed when the *spoZ-CD16671* is overexpressed in these strains. Finally, the relative abundance of SpoZ is less in R20291 than in 630∆*erm.* This data is now discussed in lines 502-521 in the Results section and lines in the Discussion 583-587.

O the authors speculate that the contradicting results might be due to differences in sRNA levels -> is it possible to to test this hypothesis by combining overexpression and gradual/partial knock-down and look at sporulation frequencies in these strains (supplemented with NB validation of sRNA expression)?

Fortunately, the recommended experiment below to overexpress CD16671 provided us with a plausible hypothesis to explain the contradicting sporulation phenotypes. Thank you! We do now include the northern blot as well, probing SpoZ in 630∆*erm*, the *rgaR* mutant, the *spoZ-CD16671* mutant, and R20291 (new **Fig. 5B**).

· Finally, I would like to suggest renaming the sRNA, since it is not the only sRNA regulating sporulation in C. difficile.

Although this regulatory sRNA impacts later stages of sporulation, we acknowledge that directly implying its involvement in *C. difficile* sporulation through its name is important. We have renamed this sRNA, SpoZ.

Regarding CD16671 (and the lack of a phenotype):

· The antisense transcription of SrsR might lead to translational inhibition or degradation of CD16671. Accordingly, the sRNA knockout could lead to increased CD16671 transcript levels. Perhaps, it would be worth testing a strain that overexpresses CD16671 (instead of a CD16671 deletion) for a potential sporulation phenotype.

This was our initial hypothesis, too, but we were misled by the absence of a sporulation phenotype in the *CD16671* mutant! To further test the potential impact CD16671 may have on *C. difficile* sporulation, we overexpressed *CD16671* in 630∆*erm* and measured sporulation frequency after 24 h of growth on sporulation agar. We found that increased *CD16671* resulted in a reduction in sporulation frequency and in transcript levels of genes encoding sporulation-associated sigma factors (new **Fig. 5E and F**). We also analyzed *CD16671* transcript levels in the R20291 background (new **Fig. 7D**) and in the *spoZ* CRISPRi knockdown strain (new Fig. **S7C**), which provided us convincing data that CD16671 transcript levels correlate with the sporulation phenotypes observed (summarized in new **Table 2**).

· CD16671 has homologs in the C. difficile genome. Could a redundant function be the reason the authors don’t see an effect on sporulation in the CD16671 KO mutant?

CD16671 has one ortholog in the *C. difficile* 630 background, *CD630_19903* (*CD19903*)*,* which shares 80% nucleotide identity and 81% amino acid identity. Using qRT-PCR, we measured transcript levels of *CD19903* in both the *rgaR* and *spoZ-CD16671* mutants (Fig. S). There was no change in *CD19903* transcript levels in the *rgaR* mutant, and a variable, but statistically significant, 1.7-fold increase of *CD19903* transcript levels in the *spoZ* mutant. It is possible that CD16671 and CD19903 share a similar or identical function in *C. difficile* sporulation, but that they are differentially regulated. These possibilities will be explored in future work. For now, the new *CD19903* data is summarized in new **Fig. S8** and discussed in lines 376-384.

Minor comments:

· Line 232: promote needs an s ->… promotes sporulation …

Thank you!

· Sporulation assay is done by treating with 28.5% EtOH for 15 min. This is a rather low EtOH concentration, is this generally enough to kill the Δerm and the R20291 vegetative cells?

We empirically determined the *C. difficile* vegetative cell resistance to ethanol (Edwards *et al.* 2016 *Front Microbiol* PMID: 27833595), and determined no vegetative cells of three different *C. difficile* strains survived exposure to 28.5% ethanol for 15 min. In addition, we always use a *spo0A* mutant control in all ethanol resistance sporulation assays to ensure there is no contamination and that all vegetative cells are effectively eliminated during the current assay (Methods, lines 712-715).

· Figure 1:

o Why does the CRISPRi knockdown work better than the complete knockout (Fig2A) in inhibiting sporulation?

We observe some variation in our sporulation frequencies over time due to unknown variables that are likely related to chamber conditions or the current lot of medium components; however, the trends, the statistical significance, and the conclusions remain unchanged.

o The CRISPRi promoter seems to be leaky, since there is a clear decrease of sporulation frequency in the uninduced control – if possible, show expression of the sgRNA with a northern blot uninduced and induced?

Yes, the *cas9* gene is under control of the *cprA* promoter, which does exhibit some activity under non-inducing conditions (see Suarez *et al*. 2013 *JBac* PMID: 23543720). The sgRNA gene is under the control of the constitutive *gdh* promoter (Muh *et al.* 2019 *JBac* PMID: 30745377), so the knockdown is based on expression and activity of the caspase gene. Figure S2 shows the transcript levels of *rgaS* and *rgaR* in the uninduced and induced conditions. Both *rgaS* and *rgaR* transcript levels are significantly decreased in the uninduced conditions, showing that even at low levels of Caspase 9 accumulation, these sgRNA can efficiently block some transcription of their targets.

o In line, it might be better to compare induced to noninduced for the significance Test.

Because P*cprA-cas9* expression is leaky, the comparison to the uninduced control is not as informative or appropriate.

o Why was the standard deviation shown in A, but the standard error in C? They should be consistent with this (across the paper) for better comparability.

We use standard deviation with sample sizes (n) of three; however, to account for the variability with greater sample numbers, we use standard error of the mean with sample sizes of four or more (n = 4+). Standard deviation demonstrates how widely scattered some of the measurements are; that is, the average amount of variability. Using standard error of the mean allows us to indicate the uncertainty around the estimate of the mean. We apply SD or SEM based on sample numbers as a standard within our lab’s statistics protocols.

· Figure 2C:

o Why is there such a huge overexpression when inserting the complementation? Since the native promoter is included, this should not be the case. Are there multiple insertions or insertions into other genes affecting sporulation [I am not familiar with the method]?

The Tn*916* transposon can insert into many different places within the chromosome, although Tn*916* has been shown to have preference for some “hot spots” around the *C. difficile* genome. Some of these sites may be more accessible for transcription than other places. Also, because the original promoter regions of *rgaS* and *rgaR* are still present in the chromosome (i.e. the clean deletions only remove the open reading frames of the genes of interest), whatever promoter elements are present are now duplicated, potentially impacting transcriptional regulation. Unfortunately, we don’t have a concrete answer for this phenomenon, but the conclusions remain the same: replacing the deleted gene reverses the sporulation phenotype of the original mutants.

o Figure description: (C) should be bold; Tukey’s multiple comparison test for Figure C instead of B?

Corrected.

· It might be worth verifying srsR expression via NB in a WT compared to a rgaS rgaR mutant to see, if the TC system is the only transcriptional activator of the sRNA in the tested conditions

We performed near-infrared northern (irNorthern) blotting and confirmed that *spoZ* is expressed in the wild-type strain. The full length of SpoZ is longer than originally predicted and similar to our PCR analyses (>500 nt). The *spoZ* transcript was not detected by irNorthern in the *rgaR* mutant or the *spoZ-CD16671* mutant. Finally, *spoZ* RNA was less abundant in the R20291 background. This data is all represented in the new **Fig. 5B**.

· Table 1: What is the cutoff for up/down regulated genes?

We demarcated the genes that are differentially expressed as those with at least a 2-fold change and with *p* < 0.05. We have now added this detail to the Table 1 legend (lines 245-247) and more details about the statistics utilized by DESeq2 to the Methods (lines 753-755).

· Figure 5B is neither mentioned nor discussed in the text.

Thank you for catching this oversight! We now discuss these data in lines 314-328 (now **Fig. 4B**).

· Figure 6B: Please correct the figure legend. According to the description the graph shows gene levels in wt, agrB1D1 deletion, and complementation, but this isn’t shown in the figure.

Corrected.

· Figure 7B, line 404: There is no reference in the text

We added the Fig. 7B reference to the text (line 475).

· Figure 7D: Include a bar graph with statistical Tests for sporulation in R20291 srsR

The statistics are included in the phase contrast images (asterisks; now **Fig. 7E**), and we have included bar graphs representing this data in the Supplementary Data (**Fig. S10A, B**) so that the variability for each biological replicate can be observed as well.

Reviewer #2: Clostridioides difficile is a major gastrointestinal pathogen. The ability of this bacterium to produce spores is important for its survival in the environment, persistence in the gut, and transmission from the environment to the gut when causing disease. Despite this importance, the regulation of C. difficile sporulation remains incompletely understood at present.

This manuscript helps to address that knowledge gap by identifying orphan response regulator RgaR as a functional partner of the orphan histidine kinase RgaS in a two component regulatory system that positively-regulates several genes, including two involved in controlling sporulation. One of those two RgaS/RgaR-regulated genes encodes AgrD1; the current study confirms previous reports that this quorum sensing peptide positively regulates C. difficile sporulation and demonstrates that this regulation occurs early in the sporulation pathway. The other RgaS/RgaR-regulated gene affecting sporulation encodes SrsR, which is a small regulatory RNA. SrsR is shown to positively affect sporulation at later stages in the process.

Overall, the study did not accomplish their original goal (to identify the kinase phosphorylating Spo0A). However, this study (with a few exceptions noted below) is scientifically sound and does identify a new two component regulatory system in C. difficile that helps to regulate sporulation. It also sheds some new insights into the regulation of C. difficile sporulation. In particular, the discovery of a new small RNA involved in regulating sporulation is interesting, although precisely how this sRNA affects sporulation will require further study. There are also some intriguing findings that identify unique aspects of the C. difficile Agr quorum sensing system.

Specific comments:

1) Line 131: please briefly describe this limited similarity between the rgaS gene and other sporulation-associated phosphotransfer proteins.

To help visualize the differences between the sporulation-associated phosphotransfer proteins and RgaS, specifically within the region possessing the catalytic histidine residue, we created an amino acid alignment and include this as a new **Fig. S1**.

2) In Fig. 1A, it might help clarity if the x axis indicated that the right panel is 70:30 + nisin.

We made this change in **Fig. 1A, C** as well as **Fig. S1A, B**.

3) In Fig. 1B, was this identity/similarity of RgaS to VirS and AgrCs distributed throughout the proteins or did it cluster preferentially to either the N-terminal membrane sensor domain or the C-terminal cytoplasmic phosphorylation domain?

There is significant similarity between RgaS and VirS/AgrCs throughout the entire length of the proteins. However, there is higher identity between the proteins in the dimerization and histidine autophosphorylation domains.

4) Line 168: it is interesting that the screen identified genes encoding two VirR orthologs, RgaR and RgbR. The rationale for focusing on RgaR is clear and adding RgbR studies to the current manuscript would be unreasonable; however, the Discussion might revisit RgbR and mention the need for future studies of this response regulator. Might RgbR also be involved in regulating sporulation in C. difficile?

The original work that created and characterized *rgaR* and *rgbR* mutants did not find any transcriptional changes between the *rgbR* mutant and the parent strain, including sporulation genes, during early stationary phase in the conditions tested (O’Connor *et al*. *Mol Microbiol* 2006). Further, while overexpression of *C. difficile rgaR* complemented a *C. perfringens virR* mutant, overexpression of *C. difficile rgbR* did not (O’Connor *et al*. *Mol Microbiol* 2006), suggesting that RgbR functions differently. We have added this detail to the Results to help inform the reader why RgbR was not pursued in our studies (lines 170, 173). So far, there is no preliminary data that suggests that RgbR has a role in *C. difficile* sporulation.

5) Relative to wild-type, did the RgaS and RgaR mutants (and other mutants made in this study) show any differences in their vegetative growth?

We did not detect any differences in the growth rates of the *rgaS*, *rgaR*, *agrB1D1*, and the *spoZ-CD16671* mutants in BHIS or TY media.

6) In Fig 2A and elsewhere, the x axis labels are sometimes challenging to follow. Suggest changing rgaS to (delta)rgaS for clarity and consistency with the Figure Legend.

We have added the delta (∆) symbol to denote mutants in all of the figures.

7) In the Fig 2 legend, bold (C) for consistency with (A) and (B); on line 202, replace (B) with (C).

Thank you for catching these errors.

8) Table 1: Agree that the data suggest that rgaR mutant preferentially affects late sporulation gene expression. However,
a. Please indicate what fold-change was considered a “significant” regulatory effect in this RNA-seq experiment?

We have now added this detail to the Table 1 legend (lines 245-247) and more details about the statistics utilized by DESeq2 to the Methods (lines 753-755).

b. It is interesting that the only upregulated genes in the rgaR mutant were Spo0A dependent. Do the authors have any thoughts about this?

Yes, several of the upregulated genes are Spo0A-dependent genes or other genes encoding early sporulation factors, which is not surprising. Late-stage sporulation mutants will have increased accumulation of early-stage sporulation transcripts (Fimlaid *et al*. 2013 *PLoS Genet* PMID: 23950727). There are additional upregulated genes that are not part of the Spo0A regulon; however, none of the upregulated genes contain a recognizable RgaR consensus sequence, suggesting that these genes are indirectly impacted by the absence of RgaR.

c. The RNA-seq data indicates that spo0A transcription was affected in the rgaR mutant- does this mutant have altered Spo0A or Spo0A-P levels?

The RNA-seq data does reveal that transcript levels of *spo0A* are elevated ~2-fold, but Spo0A-dependent gene expression (i.e. Spo0A activity) was largely unaffected (see **Table 1**). For Spo0A to activate transcription, it must be phosphorylated; therefore, a direct read-out for Spo0A~P activity is gene expression of Spo0A-dependent genes. We now provide data (new Fig. S4A), which demonstrates no change in transcript levels of *sigE*, a Spo0A-dependent gene, in the *rgaS* and *rgaR* mutants. Altogether, these data indicate that the increase in *spo0A* transcript levels does not impact Spo0A activity.

d. It is mentioned on line 375 that a prior study showed that AgrD1 promotes transcription of early sporulation genes. Does this include spo0A? If that is known, then does the AgrBD mutant, which shows reduced sporulation due to an effect early in sporulation, exhibit less Spo0A and Spo0A phosphorylation than wild-type? Answering this question is perhaps slightly off-topic for the current study but might begin to shed some mechanistic insights into the role of AgrD in regulating the early steps of C. difficile sporulation.

Yes, Ahmed *et al*. (2020 *mBio* PMID: 33443122) found that *spo0A* transcript levels were reduced ~2-fold in the *agrB1D1* mutant, but that there were greater decreases in *sigE* and *sigF* transcript levels (~10-fold). Determining the sporulation factor(s) that are impacted by AgrD1 signaling is of great interest, and elucidating whether AgrD1 signaling affects Spo0A phosphorylation directly or impacts expression or transcription of an early sporulation factor (e.g. RstA) that influences sporulation initiation will be one of the initial steps for this research.

9) Line 294: add “expression” after CD16671

Thank you; we added “transcript levels” here.

10) Line 302: add a reference after “interactions”.

Thank you for catching this omission.

11) Is Fig. 5B ever addressed in the text of the results?

Fig 5B is now referenced and is renamed Fig. 4B (lines 314-328)! Thank you for catching this oversight.

12) On print-outs it is sometimes hard to distinguish asterisks from solid dots in figures. Consider making these symbols slightly larger.

The asterisks are now embiggened.

13) In the Fig. 8 legend describe the meaning of “X” and “Y”; presumably these refer to the abundance of SrsR RNA?

In light of the new data generated during the response period, notably that higher levels of CD16671 inhibit sporulation, we’ve refined our model in **Fig. 8** and now only have X to represent the unknown SpoZ target. We’ve modified the Discussion and have added **Table 2** to summarize this new data.

14) References need some work. Please double check.
a. The style is inconsistent. Specifically, in titles of some references the first letter of every word is capitalized, while in other references the first letter of every word in the title is capitalized. PLoS Genetics presumably has a reference style and this should be adhered to. If not, then pick one style and be consistent.
b. Some references are incomplete. Fpr example, reference 12 lacks page numbers.
c. Reference 20: please correct the title of this reference. There are some extraneous symbols present.

Thank you for recognizing these errors. We have corrected and formatted the references according to PLoS Genetics’ style.

Reviewer #3: This work from Edwards and McBride provides further insight into the unique complexities of regulatory networks controlling C. difficile sporulation. Leveraging sequence similarity to other clostridial regulators of Spo0A phosphorylation they examined a cognate histidine kinase and response regulator. RgaS-RgaR was discovered to control late-stage sporulation. Suppressing expression of RgaS and/or RgaR reduced sporulation efficiency, which was recovered by complementation. They further connected this to control of a small RNA, SrsR, but were unable to dissect this regulatory RNA’s mechanism of action. They further eliminate regulation by AgrD1 yet show connections to expression of the Agr system. Finally, they show the system is conserved in other strains of C. difficile. Overall, the paper is well-written, and the experiments are rigorous. Although there is more work to be done, further investigation into SrsR will be a major undertaking. As such, I’m comfortable with leaving that portion of the study open for interpretation.

The authors are quick to conclude that RgaS-RgaR has little influence on Spo0A. What is the C. difficile Spo0A regulon? How has that been defined and reported? I’m familiar with the previous work from the Shen group, but my recollection is that study focused on sigma factors. I don’t recall a C. difficile Spo0A regulon reported in the literature and the authors don’t reference one.

Yes, our RNA-seq data in Table 1 is compiled based on work from the Shen group (Fimlaid *et al*. 2013 *PLoS Genet* PMID: 23950727) where they delineate the Spo0A regulon along with the regulons of the sporulation-associated sigma factors via RNA-seq analyses. To reduce confusion, we’ve added this detail into Table 1, Footnote A.

Is it possible the authors have discovered a more refined Spo0A regulon subject to upstream control by RgaS and/or RgaS-RgaR? One might posit RgaS phosphorylates Spo0A and transcription of SrsR tempers the rate of sporulation, maybe as a checkpoint control in sporulation. Thus, when RgaS is absent Spo0A is not activated and sporulation is reduced, regardless of SrsR activity. When srsR is deleted, Spo0A might still be phosphorylated thereby explaining the hypersporulation phenotype. This could explain the “internally inconsistent” data mentioned in the discussion. Unfortunately, the authors didn’t check for changes in Spo0A phosphorylation so it's difficult to know if this model is within the realm of possibilities. The authors should examine Spo0A phosphorylation in their knockdown strains.

We also considered the possibility that Spo0A may be directly phosphorylated by RgaS. However, the dimerization and histidine autophosphorylation domain (H-box) of RgaS diverges significantly from the known sporulation-associated phosphotransfer proteins (Fig. S1), including PtpC, which has been shown to directly phosphorylate Spo0A in vitro (Underwood *et al.* 2009 *J Bacteriol* PMID: 19783633). Overall our data are consistent that RgaS and RgaR function as a cognate two-component system. The sporulation phenotypes and gene expression profiles of the *rgaS* and *rgaR* single mutants and *rgaS rgaR* double mutant are all identical, suggesting that RgaS-RgaR function together to influence sporulation.

To further confirm that the RgaS-RgaR system does not impact Spo0A activity, we now include qRT-PCR data on *sigE*, Spo0A-dependent gene, demonstrating that there is no significant change in *sigE* transcript levels in the *rgaS* or *rgaR* mutants (Fig. S4).

Finally, new data we obtained during the review period now provide a simpler explanation for the two sporulation phenotypes (the “internally inconsistent” data) observed with *spoZ.* In strains where *spoZ* transcript levels are low and *CD16671* transcript levels are high, sporulation is inhibited (e.g. in the *rgaS* mutant, the *rgaR* mutant, when *CD16671* is overexpressed). However, in conditions where *spoZ* transcript levels are low and *CD16671* transcript levels are unchanged or lower (e.g. the *spoZ-CD16671* mutant and the *spoZ* knockdown strain), a hypersporulation phenotype is observed. This data suggests that CD16671 inhibits sporulation, which is dominant to SpoZ regulatory effects on an unknown target that likely promotes sporulation. Additional work beyond the scope of this study will need to be performed to confirm and refine our current model.
